# Multi‐Functional Polydopamine‐Mucin Hollow Particles Provide Tunable Shell Permeability, ROS Scavenging, Tissue Adhesion, and Lubricity for Biomedical Applications

**DOI:** 10.1002/smll.202503238

**Published:** 2025-07-04

**Authors:** Di Fan, Chiara Gunnella, Yukun Wang, Luca Reichert, Pedro Henrique da Rosa Braun, Jan Torgersen, Oliver Lieleg

**Affiliations:** ^1^ Department of Materials Engineering School of Engineering and Design Center for Protein Assemblies and Munich Institute of Biomedical Engineering Technical University of Munich Ernst‐Otto‐Fischer Str. 8 85748 Garching Germany; ^2^ Department of Materials Engineering School of Engineering and Design Institute of Materials Science Technical University of Munich Boltzmannstraße 15 85748 Garching Germany

**Keywords:** drug encapsulation, free radical scavenging, lubrication, tissue adhesive, wear prevention

## Abstract

Owing to their high drug loading capacity and the option to functionalize their shells, hollow particles (HPs) have emerged as versatile platforms for diagnostic and therapeutic applications. However, those two key advantages of HPs are not yet well leveraged. Often, the large volume within the shells is not fully utilized as a consequence of the employed drug loading methods, and to date, only a limited range of functionalities can be successfully implemented into the shells of HPs. Here, the self‐polymerization and adhesion behavior of dopamine are utilized, for the first time, to fabricate polydopamine (PDA)‐mucin HPs using a template‐based method. By adopting molecules or ions as “locks” to adjust the permeability of the shells, cargo molecules can be trapped within the shells with high encapsulation efficiency. Moreover, owing to the intrinsic properties of PDA and mucins, the shells exhibit multiple functionalities in vitro and ex vivo, including free radical scavenging, tissue adhesion, lubrication, and wear prevention. This study presents a facile method to produce multi‐functional PDA‐based HPs from a range of (bio)polymers, thus facilitating potential applications of HPs for the treatment of certain diseases, including osteoarthritis and mouth ulcers.

## Graphical abstract

This study presents a facile method to fabricate hollow, multifunctional polydopamine (PDA)‐mucin microparticles. The permeability of the particle shells can be tuned by adding ionic or molecular “locks”, so that cargo molecules can be trapped within the shells with high encapsulation efficiency. Moreover, such hollow microparticles can scavenge free radicals, adhere to tissues, reduce friction, and prevent surface wear.

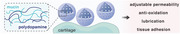

## 1 Introduction

Drug delivery systems have revolutionized the diagnosis and therapy of numerous diseases by enabling the targeted delivery of theranostic agents, reducing the non‐specific biodistribution of pharmaceuticals, extending the half‐life of drugs, and improving patient compliance.^[^ ^1^, ^2^ ^]^ Among the broad variety of delivery systems, hollow particles (HPs) have emerged as promising platforms for various biomedical applications such as the treatment of joint diseases and ulcers.^[^ ^3^, ^4^, ^5^ ^]^ Several strategies have been developed to fabricate HPs, and those can be categorized into i) template‐based methods, which employ sacrificial cores,^[^ ^6^, ^7^, ^8^ ^]^ ii) self‐templating methods, where solid particles undergo a self‐transformation into hollow structures,^[^ ^9^, ^10^ ^]^ and iii) biosynthesis methods, where the HPs are produced by bacteria or cells.^[^ ^11^, ^12^ ^]^ With those approaches, HPs can be produced from diverse materials, including inorganic components (e.g., silica and metal),^[^ ^13^, ^14^, ^15^ ^]^ organic compounds (e.g., (bio)polymers and proteins),^[^ ^7^, ^11^ ^]^ or hybrid inorganic–organic materials (e.g., metal–organic frameworks, MOFs).^[^ ^10^ ^]^

A key advantage of HPs is their high loading capacity, which originates from their high surface‐to‐volume ratio, high shell porosity, and their spacious empty cores.^[^ ^16^ ^]^ However, the volume inside the HPs is often not utilized to its full potential. Typically, cargo molecules are loaded into HPs by trapping them inside due to size exclusion effects,^[^ ^17^ ^]^ or they are anchored to the particle shells through molecular interactions.^[^ ^18^ ^]^ Conventional strategies employed for cargo trapping in HPs involve three steps: first, loading drugs onto the surface and/or into the volume of porous template particles; second, creating another coating around the drug‐coated templates; third, dissolving the cores. However, this approach does not lead to a high drug loading capacity, as the presence of the solid template cores limits the space available for cargo molecules. Moreover, this method is only feasible for cargo molecules with large molecular weights (MWs) such as proteins, thereby narrowing the range of loadable drugs. Similarly, limitations are also found when loading the drugs onto the HP shells via interactions between the shell constituents and the cargo molecules. Here, the cargo molecules need to possess suitable physico‐chemical properties to bind to the HP shell, e.g., via electrostatic forces,^[^ ^19^ ^]^ coordinate bonds,^[^ ^20^ ^]^ or *π–π* stacking.^[^ ^21^ ^]^ This requirement not only limits the range of loadable drugs but also necessitates specialized designs for the HPs to enable effective drug release. More importantly, this loading position exposes the cargos to chemical/physical challenges from the outside environment, which may cause premature degradation of the cargos (e.g., in the case of siRNA and peptides).^[^ ^22^, ^23^ ^]^ Thus, developing HPs with tunable permeability properties to allow for cargo loading within their shells offers a promising strategy to improve the drug loading capacity of the particles and to protect the cargo against external conditions.

In addition to acting as drug carriers, HPs may bring about extra benefits when their shells are endowed with desired functionalities. To make this possible, the materials constituting the shells need to be carefully selected, and possible choices include MOFs (as catalysts, imaging agents, and photoconversion agents),^[^ ^24^, ^25^, ^26^ ^]^ hydroxyapatite (for bone regeneration),^[^ ^27^ ^]^ or polyphenols (for relieving oxidative stress).^[^ ^28^ ^]^ Among those options, polydopamine (PDA), a polyphenol variant, may be of particular interest since it can be used to create HPs with functional shells by employing template‐based methods.^[^ ^29^ ^]^ Here, the self‐polymerization behavior of dopamine allows for the direct creation of PDA coatings on the surface of sacrificial template particles.^[^ ^30^ ^]^ Such PDA HPs show good UV‐absorption and near‐infrared (NIR)‐conversion capabilities, and can serve as UV‐filtering materials and NIR‐responsive drug carriers.^[^ ^31^, ^32^ ^]^ In addition, PDA can chelate metal ions to create PDA‐metal HPs, and those HPs can generate free radicals via Fenton or Fenton‐like reactions for applications in chemodynamic therapy.^[^ ^33^ ^]^ However, PDA‐based HPs might even hold greater potential beyond providing the aforementioned functionalities. For instance, PDA has been reported to be an excellent antioxidant and versatile adhesive to a broad range of materials.^[^ ^34^, ^35^ ^]^ However, those two properties of PDA have not been harnessed for PDA‐based HPs yet. Such antioxidative and adhesive properties could be beneficial for the treatment of certain diseases, e.g., osteoarthritis, chronic wounds, and inflammatory bowel disease, where oxidative stress and fast drug clearance are concerns.^[^ ^28^, ^36^, ^37^, ^38^, ^39^ ^]^ To tackle these challenges, PDA‐based HPs could adhere to the target tissue and provide both prolonged drug retention and relief of oxidative stress.

Multiple methods for fabricating PDA HPs have been developed to generate different hollow structures, such as nano‐bottles and nanotubes,^[^ ^32^, ^40^ ^]^ or to achieve different particle compositions, such as PDA‐based MOF particles.^[^ ^41^, ^42^ ^]^ However, (bio)polymers have rarely been co‐deposited with PDA to form HPs. This is somewhat surprising considering that a method to co‐deposit PDA and a broad range of (bio)polymers to create surface coatings was developed over a decade ago.^[^ ^43^ ^]^ With a similar approach, it should be possible to fabricate hybrid biopolymer‐PDA HPs, whose permeability might then be easier to control by crosslinking the polymer components; more importantly, the flexible conformations of the biopolymer chains can help to maintain the particle integrity in the presence of crosslinking‐induced forces. In addition, incorporating additional (bio)polymers into PDA‐based HPs can further extend their functionalities beyond acting as drug reservoirs only. A promising candidate for creating such multifunctional PDA‐(bio)polymer blended HPs are mucin glycoproteins (the macromolecular key components of mucus), which contain diverse chemical groups to allow for their binding to PDA; in addition, their large sizes and dense glycan side chains along the polypeptide backbone may help stabilizing the HPs.^[^ ^44^ ^]^ Since mucins exhibit high biocompatibility, low immunogenicity, antibiofouling properties, mucoadhesive behavior, and lubricating abilities,^[^ ^44^, ^45^, ^46^, ^47^, ^48^ ^]^ they are likely to provide HPs with multifunctional properties.

Owing to those interesting properties, mucins have already been used to construct particles for drug delivery purposes (yet only in the sub‐micron size range), and those nanoparticles were produced by condensing mucin molecules using a suitable solvent (e.g., glycerol or ethanol)^[^ ^48^, ^49^ ^]^ or cationic molecules (e.g., chitosan).^[^ ^50^ ^]^ After crosslinking those condensed mucins with ions, DNA strands, or covalent bonds, the obtained mucin nanoparticles exhibit prolonged stability and the ability to release drugs on demand.^[^ ^51^, ^52^, ^53^ ^]^ Still, alternative fabrication strategies for mucin‐based particles are required, given that different methods produce particles with diverse properties regarding size, charge, and stability. Additionally, since conformational changes of mucin molecules can alter their lubricating properties,^[^ ^54^ ^]^ the lubricating potential of mucins can strongly depend on the formulation (e.g., solutions, particles, coatings, or hydrogels) in which they are used. However, the lubricating properties of mucin‐based nano‐/micro‐particles have, to the best of our knowledge, not been investigated yet.

**Figure 1:**
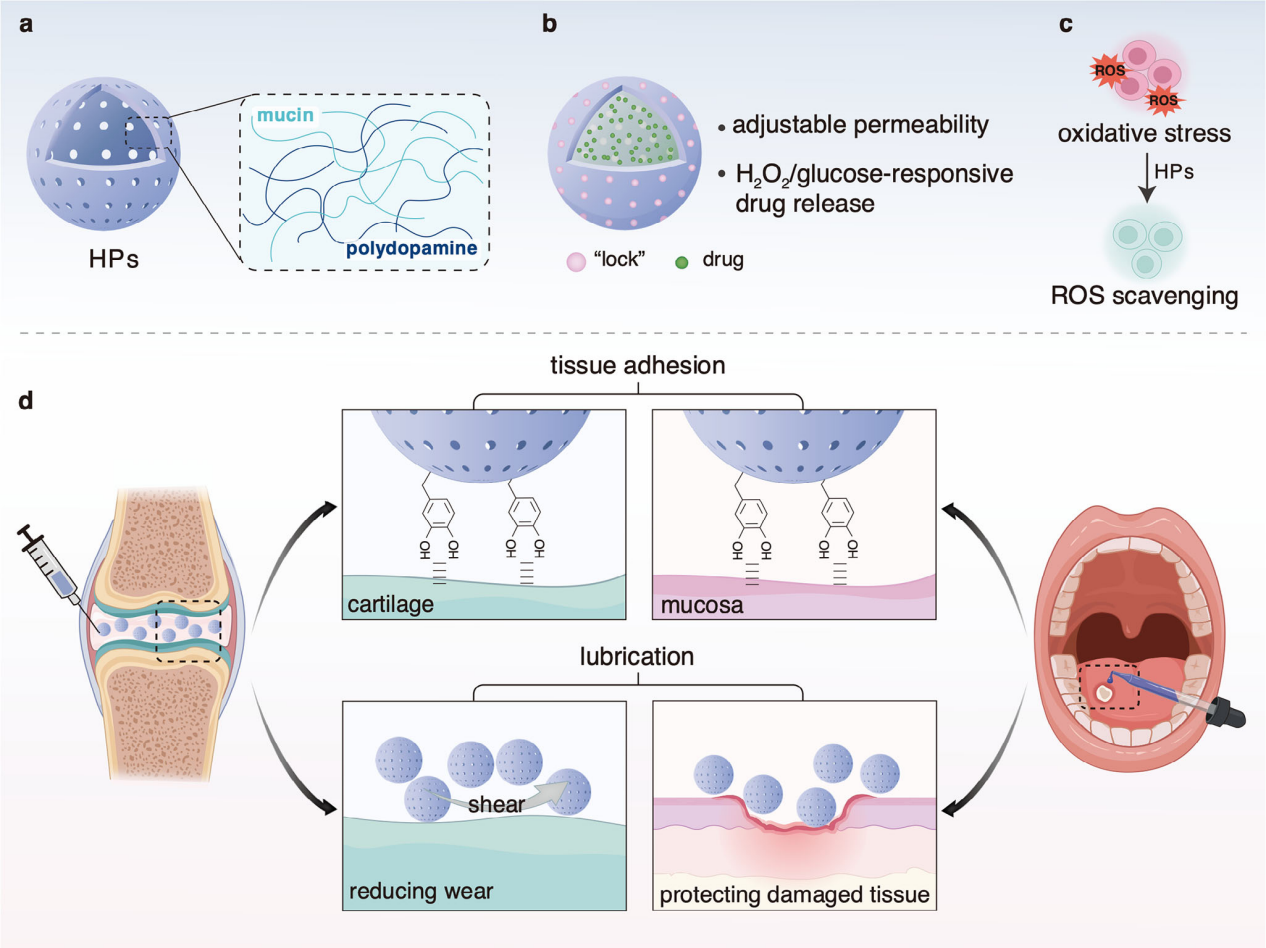
Schematic illustration of the properties and functionalities of the HPs developed here. a) The HPs are produced from a mixture of PDA and mucin macromolecules. b) The permeability of the HPs can be tuned by employing different crosslinkers for the HP shells, which “lock” the loaded cargo molecules into the HP core. The drug release behavior of the HPs responds to the presence of either hydrogen peroxide (H_2_O_2_) or glucose in the environment. c) The HPs can scavenge free radicals, which can protect cells. d) The HPs exhibit good adhesion properties to tissues, and thus could be used to treat lesions on cartilage or tongue tissue by providing a prolonged retention time for drugs and reactive oxygen species (ROS) scavenging. Moreover, the HPs have lubricating properties and reduce friction and wear, which can help protecting damaged tissue areas.

In this study, we develop multi‐functional HPs from a mixture of PDA and mucin (**Figure** **1**). By co‐depositing PDA and mucins onto a sacrificial template, i.e., calcium carbonate (CaCO_3_) microparticles, the PDA‐mucin HPs can be easily fabricated after dissolving the template cores. The permeability of the obtained HPs to the cargo molecules can be efficiently adjusted by employing different “locking” agents (molecules or ions), making it possible to trap cargo molecules in the volume of the hollow cores with high encapsulation efficiency. Moreover, we examine the cargo release profiles of Ag^+^‐“locked” HPs, which show different release kinetics when exposed to hydrogen peroxide (H_2_O_2_) and glucose, respectively. Importantly, our HPs demonstrate excellent free radical scavenging abilities and can, in the presence of H_2_O_2_, either protect or kill cells – depending on whether Ag^+^ ions are used as “locking” agents or not. Ex vivo tests with porcine cartilage and tongue tissue samples demonstrate the excellent adhesive properties of the HPs. Tribological measurements illustrate the lubricating properties of the HPs, which can also reduce the formation of tissue damage on articular cartilage surfaces. Overall, the method we propose here to fabricate HPs from PDA‐biopolymer blends allows for creating drug carrier objects with well‐defined sizes in the micron range that exhibit adjustable permeability for drug loading and highly functional shells.

## 2 Results and Discussion

### 2.1 Production and Characterization of HPs

**Figure 2:**
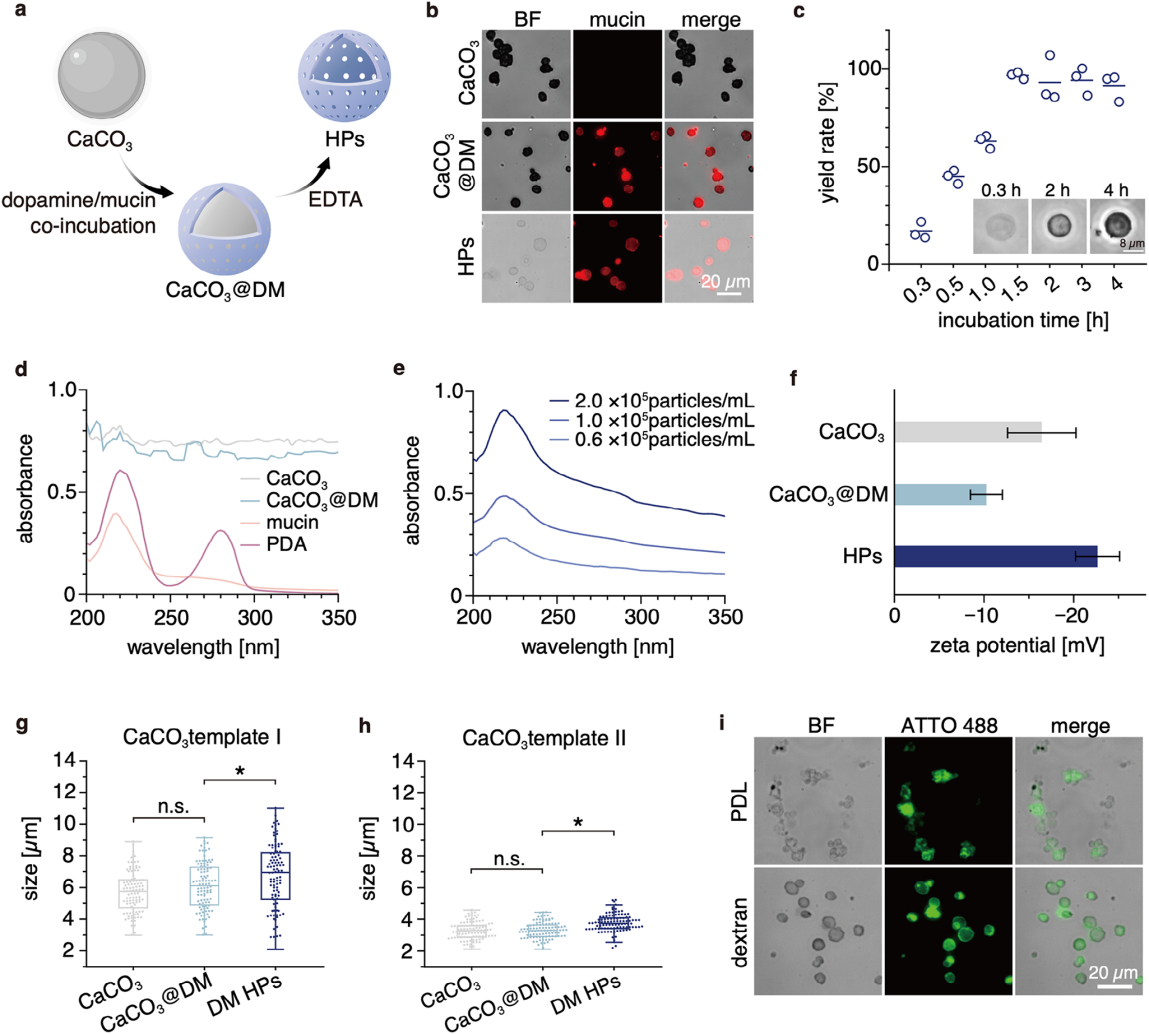
Fabrication and characterization of the HPs. a) To produce the HPs, dopamine and mucin are co‐incubated with porous CaCO_3_ microparticles, followed by an EDTA treatment to dissolve the CaCO_3_ cores. b) Bright field (BF) and fluorescence images of the particles (in ddH_2_O), where the mucin molecules are covalently labeled with ATTO 590 (red signal). c) Yield rate of the HPs as a function of the incubation time of the dopamine/mucin mixture with the CaCO_3_ templates (*n* = 3). The inset shows exemplary phase contrast images of HPs obtained after different incubation times. d) Absorbance spectra of solutions containing different particles or molecules in ddH_2_O or e) different concentrations of the HPs in ddH_2_O. f) Zeta potential of the particles in 10 mm HEPES buffer (pH 7.4), *n* = 3. The data is expressed as mean ± standard deviation. g,h) Size distributions of the particles (in ddH_2_O) when using different CaCO_3_ templates (template I: average size ≈6 µm; template II: average size ≈3 µm), *n* = 100. i) HPs made from PDA and other polymers (PDL or dextran) imaged in BF and fluorescence mode. The polymer component is covalently labeled with ATTO 488 (green signal). Asterisks (^*^) mark statistically significant differences determined in two‐tailed t‐tests based on a *p*‐value of 0.05; “n.s.” denotes non‐significant differences.

To produce HPs, we employ a process based on sacrificial templating: first, calcium carbonate (CaCO_3_) microparticles are incubated with a mixture of dopamine and mucin to obtain PDA‐mucin (DM)‐coated CaCO_3_ (named CaCO_3_@DM); second, the template core is dissolved by adding ethylenediamine tetraacetic acid (EDTA) (**Figure** **2**a). We expect that the DM coatings can be directly generated on the calcium particles via a simple co‐incubation step at pH 8.5, where the dopamine molecules can self‐polymerize into PDA and thus serve as a “sticky” component to anchor the mucin molecules to the CaCO_3_ microparticles (see Figure S1, Supporting Information) for structural schematics of PDA and mucin, respectively). And indeed, the color of the CaCO_3_ particles changes from white to black after this co‐incubation step with DM (Figure S2, Supporting Information), indicating the successful attachment of PDA to the microparticles. The presence of mucins in the coating is verified using fluorescence microscopy: after the co‐incubation step with DM, almost all particles carry fluorescent coatings which originate from the fluorescently labeled mucins (Figure 2b). Additionally, the binding between PDA and mucin is confirmed via a quartz crystal microbalance with dissipation monitoring (QCM‐D) measurement (Figure S3, Supporting Information). Having demonstrated the robustness of the coating procedure, we next ask if the DM coatings remain intact after removing the CaCO_3_ cores with EDTA – this would result in hollow structures. As anticipated, we find intact HPs (Figure 2b) where the solid (=  black) cores have disappeared when imaging them in bright field (BF) mode, whereas the fluorescence signals from the (labeled) mucins are maintained (zoomed‐out images of the particles shown in Figure 2b are shown in Figure S4, Supporting Information). Tracking the dissolving process of the CaCO_3_ cores in the presence of EDTA (Figure S5, Supporting Information) under the microscope shows a similar result: the dark cores become transparent over 37 min, whereas the polymer shells persist.

Next, we vary the co‐deposition time of DM to obtain a high yield rate (i.e., the ratio of the obtained amount of HPs to the amount of CaCO_3_ particles used) for the HPs. Here, our rationale is that the polymerization time of dopamine can influence the cross‐linking strength between distinct mucins and thus the stability of the HPs. As shown in Figure 2c, higher co‐incubation times of DM result in darker HPs (see inset) and higher particle yield rates: we can strongly increase the yield rate of the HPs from 17% to 97% by changing the incubation time from 15 min to 1.5 h. Moreover, we find that the median thickness of the particle shells becomes larger with longer incubation times (increasing from 0.63 µm at 1.5 h of incubation to 0.86 µm at 4 h of incubation; Figure S6, Supporting Information), which could result from the time‐dependent self‐polymerization of dopamine. In addition, the thickness of the shells is not significantly different for incubation times of 0.5 h and 1.5 h, even though the corresponding particle yield rates are different. This indicates that a minimum shell thickness is required to form stable HPs. For co‐incubation times above 1.5 h, the yield rate does not improve anymore; instead, more black nano‐/micro‐particles (formed by PDA) appear in the background of the sample, thus reducing the sample purity. Therefore, a co‐incubation time of 1.5 h is selected for the remainder of this study. Moreover, such obtained HPs exhibit a low density with a median value of 0.69 mg mm^−3^ (Figure S7a, Supporting Information), which can be attributed to the hollow structure of the particles. Within the HPs (which contain PDA and mucin molecules), the mass concentration of mucin is estimated to be 11 ± 1 % (w/w) (Figure S7b, Supporting Information). Of note, the stability of such prepared HPs is very good, as we do not find obvious changes in the concentration and size distribution of the particles for at least 14 days (Figure S8, Supporting Information) when the HPs are incubated either under simulated physiological conditions (i.e., in simulated synovial fluid at 37 °C) or at storage conditions (i.e., in ddH_2_O at 4 °C).

The spectroscopic properties of the particles and the molecules used to fabricate them are summarized in Figure 2d,e. Owing to the strong light scattering effect of solid microparticles, the intrinsic absorption properties of the molecules comprising the particles tend to be overwhelmed.^[^ ^55^ ^]^ Thus, no absorption peak in the UV range is observed for both, CaCO_3_ and CaCO_3_@DM microparticles. After dissolving the CaCO_3_ cores, however, the light scattering effect becomes less prominent: now, the HPs show a clear absorption peak at 220 nm, whose height depends on the particle concentration. This peak position agrees with the main absorption peaks detected for both, mucins and PDA, which constitute the shell of the HPs. In addition, a minor shoulder‐like peak at ≈280 nm is observed for the HPs, which could result from the second peak (at 280 nm) of PDA. However, this shoulder peak of HPs is not very clear, as the light scattering effect still exists for the HPs (even though it is less pronounced compared to the effect brought about by CaCO_3_ particles). Next, we conduct Fourier transform infrared (FTIR) spectroscopy to confirm the removal of the template CaCO_3_ (core) and to verify the presence of PDA and mucin in the HP shells. The CaCO_3_ spectrum shows characteristic carbonate vibrations (Figure S9, Supporting Information): out‐of‐plane bending at 874 cm⁻¹, in‐plane bending at 712 cm⁻¹,^[^ ^56^ ^]^ and vibrational bands at 1394 cm⁻¹.^[^ ^57^ ^]^ These peaks are also present in the CaCO_3_@DM spectrum, indicating that the organic coatings (PDA and mucin) have minimal spectral impact due to the dominance of the core material (v/v). After removing the CaCO_3_ cores, the carbonate peak at 874 cm⁻¹ is strongly reduced in the HP spectrum, confirming the effective dissolution of CaCO_3_. Moreover, the HP spectrum reveals a broad band from 3696–1777 cm⁻¹ (attributed to O–H and N–H stretching), which appears in both, the PDA and mucin spectra. In particular, the HP spectrum exhibits a peak at 1444 cm⁻¹, consistent with the PDA spectrum; and peaks of the HP spectrum at 1630 and 1540 cm⁻¹ (amide I and II bands), 1373, 1311, and 1033 cm⁻¹ (a strong C–O stretch) also exist in the mucin spectrum.^[^ ^58^, ^59^ ^]^ These observations confirm the presence of mucin and PDA in the HP shells.

The zeta potential of the particles is next examined since this property will influence how the particles interact with their environment and indicates the colloidal stability of a dispersion.^[^ ^60^ ^]^ As shown in Figure 2f, all particle variants exhibit negative zeta potentials. Applying the DM coating reduces the zeta potential of the CaCO_3_ particles (from ‐16 mV to ‐10 mV), which agrees with previous surface zeta potential results obtained for DM coatings applied to the bottom of well plates.^[^ ^61^ ^]^ However, we measure a very strongly negative zeta potential (−22 mV) for the DM HPs, which is somewhat surprising considering that they lack the strongly anionic core. Possibly, this result can be explained by the much lower density of the HPs compared to the CaCO_3_ and CaCO_3_@DM particles, which leads to a higher velocity of the HPs in an oscillating electrical field and thus to a higher (apparent) zeta potential (based on the Henry Equation).^[^ ^62^ ^]^

Other polymer‐based HPs (made from polycations and polyanions) generated by a similar templating approach have previously been reported to shrink or even form solid microgels after core dissolution.^[^ ^7^, ^63^ ^]^ Thus, we next ask if the size of the HPs generated here is stable after removing the CaCO_3_ template. Interestingly, we find that our DM HPs become slightly larger once their cores are removed. As shown in Figure 2g, adding DM coatings to the CaCO_3_ particles does not result in a change in the particle size, whereas the core dissolution significantly increases the average particle size from ≈6.1 to ≈7.0 µm. When we employ smaller CaCO_3_ particles (with a median size of 3.3 µm) as a template for the same fabrication process (Figure 2h), the size of the particles is increased to 3.8 µm after core dissolution – and this corresponds to a similar relative size increase as observed for the larger template. The observed difference in size after core removal could be due to intrinsic polymer properties (e.g., molecular weight, charge density, and hydrophilicity) and the interaction forces between the polymers within the shells, thus further influencing the surface tension of the HPs.^[^ ^7^, ^63^, ^64^ ^]^ More specifically, the negatively‐charged PDA and mucin molecules are likely to experience electrostatic repulsion forces,^[^ ^65^, ^66^ ^]^ which are balanced by an “anchoring” force provided by the core particle that they bind to. Then, after core removal, the repulsive forces within the shell would become more prominent, thus inducing a slight swelling of the particles.

Having characterized the HPs generated from a dopamine/mucin mixture, we ask if this PDA‐mediated fabrication method can be extended to generate similar HPs from other polymers as well. To do so, we select two polymers with different net charges, i.e., cationic poly‐D‐lysine (PDL) and uncharged dextran, both of which are labeled with the fluorophore ATTO 488. As demonstrated in Figure 2i, the dopamine‐based process allows us to successfully produce HPs from either macromolecule. However, the PDA‐PDL HPs exhibit more irregular shapes compared to PDA‐mucin or PDA‐dextran HPs, which can be a result of strong electrostatic forces acting between PDA and PDL. Nonetheless, these results indicate that the dopamine‐based fabrication method employed here is very suitable for anionic polymers (such as mucins) or uncharged polymers (such as dextrans). Interestingly, we note that the size of the dextran HPs is not significantly different from that of the core particles (Figure S10, Supporting Information). This result could be due to the low level of electrostatic repulsion between the molecules within the PDA‐dextran shells, which agrees with our notion that DM shells swell after removing the core due to electrostatic effects.

### 2.2 Employing “Locks” to Encapsulate Cargo Molecules into the HPs

**Figure 3:**
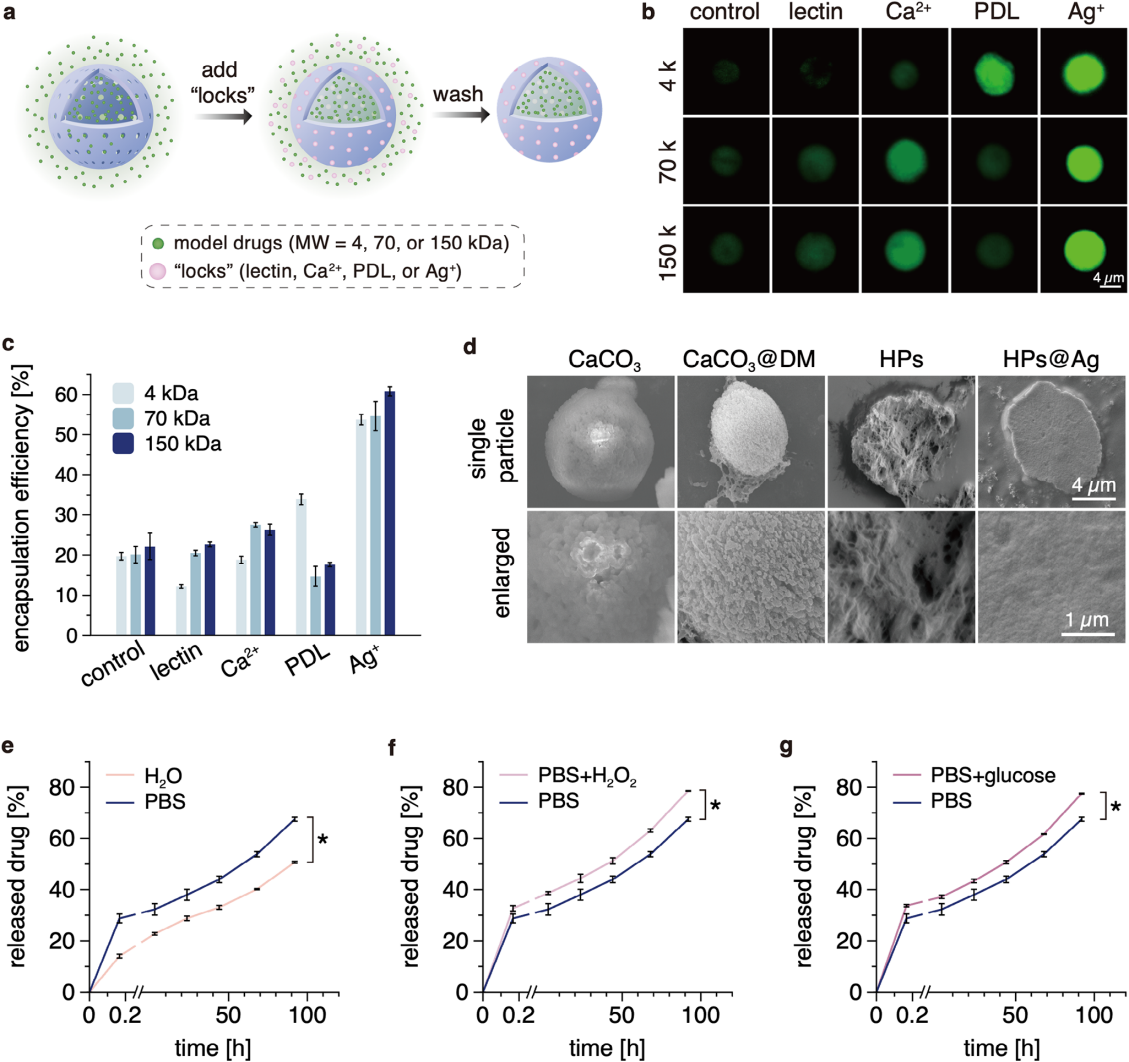
Loading and release behavior of the HPs when employing different molecular/ionic “locks”. a) Schematic illustration of the steps used to trap cargo molecules in the HPs by crosslinking the shell of the HPs. b) Fluorescence images of the HPs after cargo loading; the range of tested “locks” includes lectin, calcium ions, PDL, and silver ions. FITC‐labeled (green signal) CM‐dextrans with different molecular weights (4, 70, 150 kDa) are used as model cargos. In the control group, no locks are used. c) Encapsulation efficiency of different CM‐dextran variants into HPs when using different “locks” (*n * =  3). d) SEM images of the particles. The scale bars shown apply to all images in the respective row. e–g) Release profiles of CM‐dextran (MW  =  4 kDa) from HPs@Ag; data obtained in ddH_2_O, PBS (pH 7.4), H_2_O_2_ (10 mm in PBS; pH 7.4), and glucose (8 mg mL^−1^ in PBS; pH 7.4) is compared (*n * =  3). The data is expressed as mean ± standard deviation. Asterisks (^*^) mark statistically significant differences determined in two‐tailed *t*‐tests based on a *p*‐value of 0.05.

For the particles developed here, we expect that cargo molecules can be loaded to the HPs either by leveraging physicochemical interactions between the cargos and the HP shells (e.g., electrostatic forces, hydrophobic forces, or covalent linkages with the particle constituents), or by confining the molecules within the core of HPs due to size exclusion effects. Accordingly, a suitable encapsulation strategy should be chosen based on the detailed properties of the drug molecules and the HPs. For positively charged cargos, our HPs can load model molecules (DEAE‐dextrans) with different molecular weights (MWs) mediated by electrostatic forces (Figure S11, Supporting Information). For negatively charged cargos, which cannot engage in attractive electrostatic interactions with the HPs, it has been reported that the cargo molecules can be first loaded onto the CaCO_3_ core particles, after which the polymer shells are added and the core is dissolved.^[^ ^17^ ^]^ However, this drug loading procedure only works well when the cargo molecules are larger than the pore size of the HPs, limiting the choices of possible drug molecules that can be integrated into the particles. Thus, we here propose a loading method that allows us to flexibly tune the permeability of the HPs toward anionic cargo molecules with sizes smaller than the pore size of the HPs. Here, the cargo molecules are allowed to enter the HPs by diffusion (Figure S12, Supporting Information), after which molecular/ionic “locks” are added to the system to reduce the permeability of the particle shells, thus trapping the cargo molecules inside the HPs (**Figure** **3**a).

To test this concept, we choose fluorescein isothiocyanate‐labeled carboxymethyl‐dextrans (FITC‐CM‐dextrans) with three different MWs as anionic model cargos and test their loading efficiencies into the HPs when using different “locks”. The “locks” are selected such that they can adjust the permeability of the HPs due to their reported interactions with PDA and/or mucins, i.e., they are expected to serve as crosslinkers for the PDA/mucin network to tune the porosity and thus the molecular weight cutoff (MWCO) of the particle shells. Based on those considerations, four “lock” options are chosen including lectin, calcium ions, PDL, and silver ions. As summarized in Figure 3b, fluorescence microscopy images demonstrate that the cargo loading/trapping abilities of the HPs can indeed be tuned by these “locks”, and we find the highest locking efficiency for the silver ions. When quantifying the encapsulation efficiency of the different FITC‐CM‐dextran variants, we obtain a similar result: silver ions enhance the encapsulation efficiency the most, i.e., from 20% (obtained for HPs without using “locks”) to ≈55% (Figure 3c). Such a high encapsulation efficiency (when using Ag^+^) is comparable to that of other high‐performance drug delivery systems using FITC‐CM‐dextrans as model cargos.^[^ ^67^, ^68^, ^69^, ^70^ ^]^ In addition, our HPs@Ag exhibit a higher loading capacity of up to 70 wt.% (Figure S13, Supporting Information) than the systems reported in those studies, demonstrating the great potential of HPs@Ag to serve as drug carriers. In contrast, the selected lectin does not enhance the encapsulation efficiency of the HPs, even though the lectin variant used here is able to bind to MUC5AC mucin molecules.^[^ ^71^ ^]^ Indeed, for the 4 kDa FITC‐CM‐dextran variant, we even find a reduced loading efficiency in the presence of this lectin compared to the HPs alone. One explanation for this somewhat surprising result could be that the lectin (which binds to the carbohydrates on the mucins with high affinity and has successfully been used to cross‐link mucins in multilayer‐film assemblies)^[^ ^72^, ^73^ ^]^ competes with PDA for mucin binding sites, thus enhancing the permeability of the shells.

When using Ca^2+^ as a “locking” agent, we find a lower encapsulation efficiency for the 4 kDa CM‐dextran variant than for the larger CM‐dextrans (Figure 3b,c). This indicates that the MWCO of the HP shell as provided by the Ca^2+^ locks is in the range between 4 and 70 kDa. In general, one might assume that cargos with higher MWs can be trapped inside the “locked” HPs with higher encapsulation efficiency than smaller cargos. However, when we employ PDL as a “locking” agent, we observe the opposite trend: now, the CM‐dextran with the smallest MW (4 kDa) exhibits the highest encapsulation efficiency. Probably, at this condition, the cationic PDL macromolecules also bind strongly to the cargo molecules in the particle solution, thus reducing the amount of available PDL to “lock” the shells of the HPs. Since the net charge of the smaller 4 kDa CM‐dextrans is lower than that of their large counterparts (as larger CM‐dextrans carry more repeating units containing charged groups), such binding interactions between PDL and CM‐dextrans should be weaker here, thus leaving more PDL available for “locking” the shells and resulting in a higher encapsulation efficiency.

Since silver ions entail the highest encapsulation efficiency of the HPs, we continue with this particular “locking” agent for the remainder of this study (the Ag^+^‐“locked” HPs are named HPs@Ag). To shed light on the mechanism by which silver ions help to load more cargo molecules into the HPs, the surface morphology of the particles is visualized using a scanning electron microscope (SEM). As shown in Figure 3d, the DM coating itself (as deposited onto the CaCO_3_ particles) exhibits a porous structure. After removing the core, the pores on the HP shells appear to become somewhat larger (please note that the image depicts a collapsed particle, which results from the drying process applied to the hollow structure as required for imaging). In contrast, HPs@Ag exhibit no obvious pores on their shells, and this agrees with our observation that the permeability of the shells has been reduced by the Ag^+^ ions, as indicated by the high cargo encapsulation efficiency of HPs@Ag (Figure 3c). This reduced porosity of the HPs@Ag could be due to interactions of silver ions with PDA and/or mucins on the HPs. This hypothesis is based on reports that silver ions can bind to the catechol groups and amine groups of PDA,^[^ ^30^, ^74^ ^]^ as well as to the thiol groups of mucins.^[^ ^75^, ^76^ ^]^ To confirm which molecule(s) silver ions can bind to, we examine the size distributions of PDA and mucin molecules when co‐incubated with Ag^+^ or with Na^+^ using dynamic light scattering (DLS) measurements. Here, Na^+^ is used as a control group to account for the influence of the ionic strength on the size distribution of the molecules. As shown in Figure S14 (Supporting Information), PDA becomes larger when incubated with Ag^+^; meanwhile, PDA solutions become much darker when mixed with Ag+, indicating further oxidation of PDA caused by Ag^+^. In contrast, mucin molecules exhibit similar size distributions when mixed with Na^+^ or with Ag^+^, indicating that Ag^+^ has no strong influence on the molecular conformation/aggregation behavior of mucin. This lack of reaction of mucins to Ag^+^ ions could be attributed to the location of thiol groups on the mucin chains: the cystine‐rich domains (which contain thiol groups, Figure S1b, Supporting Information) on the peptide backbone of mucin are mostly masked by oligosaccharide side chains, and this makes it difficult for them to act as crosslinking sites. Therefore, based on these observations, we speculate that the reduced porosity of the HPs@Ag primarily results from interactions between PDA and silver ions. In particular, it is likely that PDA is further oxidized by the silver ions, thus promoting the polymerization of PDA within the particle shells; and this, in turn, can be responsible for reducing the pore size of the PDA‐based polymer matrix (see Figure S1a, Supporting Information for an overview of the potential polymerization processes of dopamine into PDA). On the other hand, silver ions can be reduced by PDA and form silver nanoparticles on the particle surfaces.^[^ ^77^, ^78^ ^]^ However, in our SEM images, we do not observe any silver nanoparticles on HPs@Ag. This could be due to the low silver concentration and short reaction time used here, which are not sufficient for generating measurable silver nanoparticles under our conditions.

Next, we investigate the drug release profiles of HPs@Ag in different solutions; for those tests, we employ CM‐dextran with an MW of 4 kDa as a model cargo. At all conditions tested here, the HPs@Ag exhibit an initial, burst‐like release within 10 min, which is followed by a sustained release phase over 92 h (Figure 3e–g). We attribute the initial burst release to the rapid detachment of loosely‐bound CM‐dextrans from the shells of the particles. In PBS (pH 7.4, 37 °C), the HPs@Ag release 67% of their cargo within 92 h (Figure 3e), which is significantly higher than the released amount determined in water (51% within 92 h). This difference could be due to ions from the buffer influencing the stability of the molecular crosslinks within the shell: a higher ionic strength can weaken the electrostatic forces acting between PDA and mucins and thus increase the permeability of HPs@Ag.^[^ ^7^ ^]^

Considering the redox‐active properties of silver ions, PDA, and mucins, we hypothesize that the drug release profile of the HPs@Ag might be responsive to oxidative and/or reductive environments, and such particular chemical conditions can be related to a range of diseases. For example, oxidative stress is a prominent feature for cancer, osteoarthritis, and inflammatory bowel disease;^[^ ^79^, ^80^, ^81^ ^]^ whereas high glucose (which is a reducing agent) levels in the blood are associated with diabetes.^[^ ^82^ ^]^ To test for a putative responsiveness of the release behavior of HPs@Ag, PBS (pH 7.4) is supplemented with either H_2_O_2_ or glucose to simulate an oxidative and reductive environment, respectively. In the presence of H_2_O_2_, HPs@Ag indeed release significantly more CM‐dextran (79%) after 92 h than in pure PBS (67%) (Figure 3f). Similarly, CM‐dextran is released at a higher rate in glucose‐supplemented PBS than in pure PBS (Figure 3g). We speculate that the silver ions play an important role in regulating the speed of the cargo release process. When exposed to H_2_O_2_, the catechol groups of PDA can be further oxidized, which makes it more difficult for silver ions to bind to them,^[^ ^83^ ^]^ therefore weakening the intermolecular crosslinks in the shells. In the presence of glucose, the silver ions might get reduced,^[^ ^84^ ^]^ thus lowering the concentration of silver ions available to crosslink the PDA‐mucin network and entailing a faster cargo release.

### 2.3 Free Radical Scavenging Abilities of HPs and HPs@Ag

**Figure 4:**
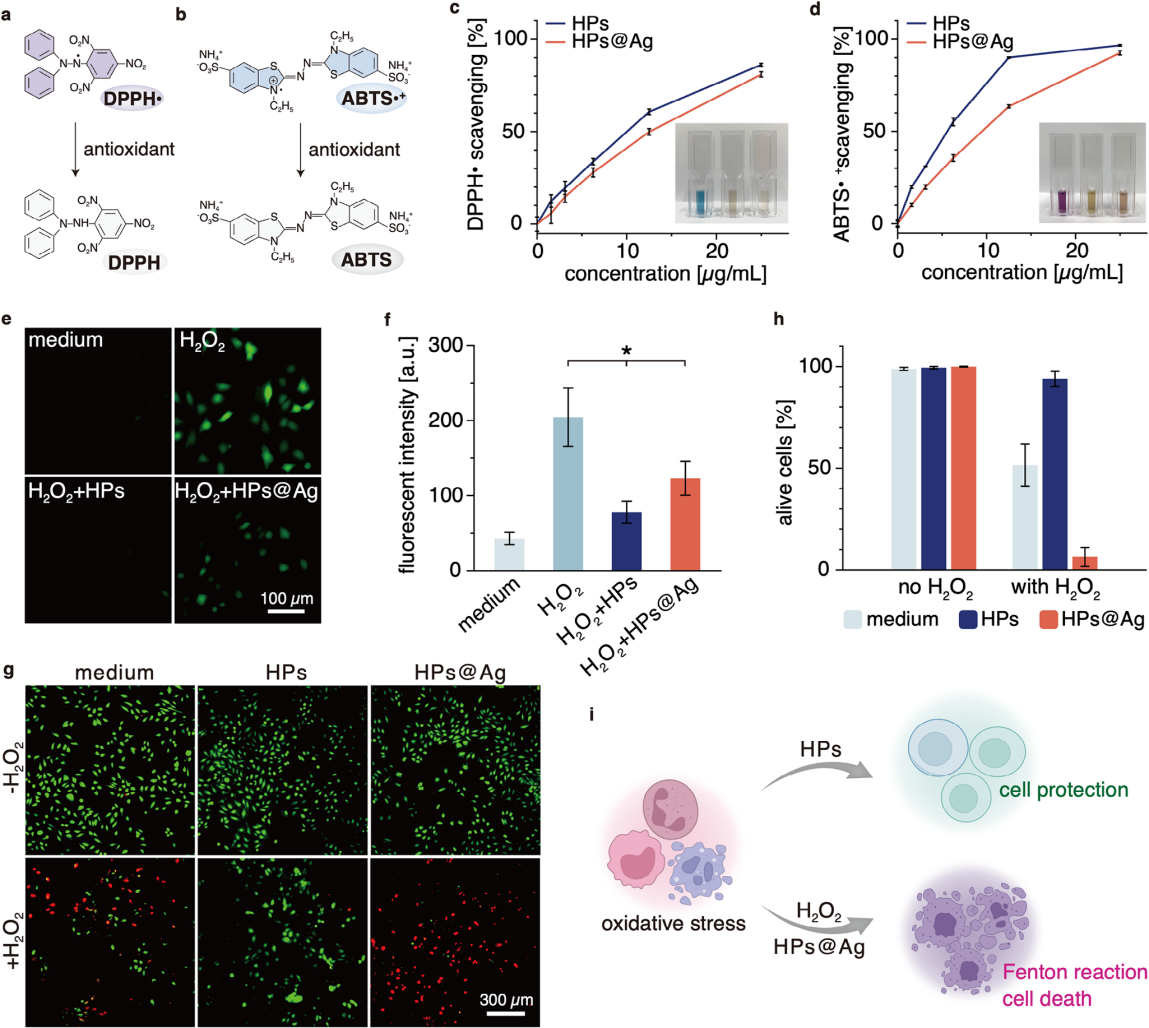
Free radical scavenging functionality of the HPs and their effects on HeLa cells. a,b) Chemical structures of DPPH• and ABTS•^+^ before and after their reaction with an antioxidant. c,d) DPPH• and ABTS•^+^ scavenging efficiency of HPs or HPs@Ag at different particle concentrations (*n* = 3). The inset shows example images of the corresponding free radical solutions mixed with water (left), with HPs (middle), and with HPs@Ag (right), where the final concentration of the particles is 25 µg mL^−1^. e) Images of the HeLa cells stained with the dye DCFH‐DA to visualize intracellular ROS (green signal). The cells are treated with medium only or with medium containing either 400 µm H_2_O_2_, 400 µm H_2_O_2_ and 100 µg mL^−1^ HPs, or 400 µm H_2_O_2_ and 100 µg mL^−1^ HPs@Ag. f) Quantification of the fluorescence intensity of the cell images after ROS staining (*n* = 4). g) Live/Dead staining results of HeLa cells treated with medium containing H_2_O_2_ (0 or 10 mm) and particles (0 or 50 µg mL^−1^); living cells appear green and dead cells appear red. h) Percentage of living cells as determined from Live/Dead staining images (*n * =  3). i) Schematic illustration of the different effects the HPs and HPs@Ag can have on the cells. The data is expressed as mean ± standard deviation. Asterisks (^*^) mark statistically significant differences determined in two‐tailed *t*‐tests based on a *p*‐value of 0.05.

In addition to possessing adjustable drug loading capability, we anticipate that our HPs can scavenge free radicals to achieve therapeutic effects. This notion is based on reports from the literature that both molecules, PDA and mucins, have antioxidative properties.^[^ ^85^, ^86^ ^]^ To test this notion, we employ two radical scavenging assays which are based on 1,1‐diphenyl‐2‐picrylhydrazyl (DPPH•) and 2,2′‐azino‐bis(3‐ethylbenzothiazoline‐6‐sulfonic acid) diammonium salt radical cations (ABTS•^+^), respectively. The DPPH• radical solution exhibits a purple color with an absorbance peak at 517 nm, whereas the ABTS•^+^ solution exhibits a blue color with an absorbance peak at 734 nm; however, after their chemical reduction, both radicals become colorless, which is accompanied by a decrease in their absorbance (**Figure** **4**a,b).

As expected, both HPs and HPs@Ag exhibit excellent DPPH• scavenging capability in a concentration‐dependent manner (Figure 4c). At a particle concentration of 25 µg mL^−1^, we find a scavenging efficiency of 86% for HPs and 81% for HPs@Ag. Similarly, at this particle concentration, HPs and HPs@Ag also possess an excellent ABTS•^+^ scavenging ability (Figure 4d), reaching 97% and 93%, respectively. Motivated by those encouraging results, we next ask which molecular component of the HPs is responsible for this free radical scavenging function, and we separately test the scavenging efficiency of PDA and mucin solutions. Interestingly, we find that, in a concentration range from 1.6 to 25 µg mL^−1^, only PDA can scavenge DPPH• and ABTS•^+^, whereas the scavenging activity of mucins is virtually zero for either radical variant (Figure S15, Supporting Information). This indicates that PDA is the main component in the HPs responsible for the observed radical scavenging effect, whereas the mucin concentrations used here (<25 µg mL^−1^) are too low to effectively reduce free radicals. Indeed, as reported in other studies, a scavenging effect of mucins is detectable when the mucin concentration exceeds 1 mg mL^−1^.^[^ ^85^, ^87^ ^]^ Of note, pure PDA solutions exhibit a higher free radical scavenging efficiency than the PDA‐based HPs created at the same total mass concentrations (w/v). This difference not only originates from the “impurity” of HPs (as mucin molecules also contribute to the weight of HPs), but also results from different oxidation levels of PDA^[^ ^88^, ^89^ ^]^ in the solution and in the HPs (as their fabrication conditions are different).

Next, we test if the radical scavenging effects of HPs and HPs@Ag can reduce the level of reactive oxygen species (ROS) in HeLa cells under chemical stress. For this purpose, the cells are treated with H_2_O_2_ to increase their intracellular ROS levels, which can be visualized by staining them with 2′,7′‐dichloro‐dihydrofluorescein diacetate (DCFH‐DA). When the cells are incubated with H_2_O_2_ in the absence of any HPs, a strong fluorescence signal is observed, whereas cells treated with medium only do not show such strong fluorescence (Figure 4e). Importantly, both HPs and HPs@Ag significantly reduce the cellular ROS level when co‐incubated with cells and H_2_O_2_; in either case, lower fluorescence signals are detected compared to the H_2_O_2_‐treated group, which has not received any particles (Figure 4e,f). We find that HPs exhibit better ROS scavenging capabilities than HPs@Ag, which agrees with the results obtained from the DPPH• and ABTS•^+^ assays. In addition, we also examine the intracellular scavenging capacity of pure PDA and pure mucin solutions, which return similar results as the DPPH• and ABTS•^+^ assays: PDA reduces the ROS level whereas mucin does not (Figure S16, Supporting Information).

Having demonstrated the promising free radical scavenging functions of the particles, we next ask whether they can protect cells from prolonged H_2_O_2_ stress. To do so, the HeLa cells are treated with a mixture of the particles and H_2_O_2_ for 5 h and then stained with Live/Dead staining solutions. As shown in Figure 4g, at the conditions chosen here, around half of the cells are dead when they are exposed to H_2_O_2_ only. In the presence of HPs, however, the percentage of living cells is increased to 94% (Figure 4h). Interestingly, the presence of HPs@Ag in combination with H_2_O_2_ strongly promotes cell death instead of protecting them. This is particularly noteworthy as HPs@Ag only (without the addition of H_2_O_2_) do not evoke cytotoxic effects. One explanation for this surprising result could be that a Fenton‐like reaction takes place between the silver ions and H_2_O_2_, and that this reaction generates hydroxyl radicals (•OH), which are lethal to the cells.^[^ ^90^, ^91^ ^]^ Such Fenton (or Fenton‐like) reactions have been applied in targeted cancer therapies (known as chemodynamic therapy, CDT), since H_2_O_2_ is overexpressed in the microenvironment of certain tumors.^[^ ^92^ ^]^ However, such reactions between metal ions and H_2_O_2_ are typically reported to be insufficient to kill the cancer cells.^[^ ^92^, ^93^ ^]^ We speculate that, here, PDA can significantly enhance the Fenton‐like reaction efficiency,^[^ ^94^ ^]^ thus creating a lethal effect brought about by the HPs@Ag in combination with H_2_O_2_. At first glance, this picture seems to be at odds with our finding that HPs@Ag (in the presence of H_2_O_2_) do not increase the ROS signals in HeLa cells (Figure 4e). However, this discrepancy might be explained by reports from the literature, which describe that a DCFH‐DA staining is not applicable to dead cells, since they cannot keep the required dye localized in the cytosol.^[^ ^95^ ^]^

Taken together, these results confirm the excellent antioxidant properties of the HPs and the possibility to adjust the function of their shells by employing different molecular/ionic “locks”. By themselves, HPs can reduce excessive levels of free radicals, which can be beneficial for the treatment of a range of diseases (e.g., osteoarthritis and chronic wounds).^[^ ^37^, ^96^ ^]^ In contrast, when “locking” the same HPs with Ag^+^ ions, the obtained HPs@Ag have the opposite effect, which might be used to specifically kill cells in an H_2_O_2_‐rich environment (e.g., in the context of a tumor‐targeted therapy).^[^ ^97^ ^]^ In other words, the option of employing slightly different variants of the same base HPs, i.e., with/without Ag^+^ “locks” (and potentially with other molecular “locks” as discussed above) creates versatile application possibilities (as illustrated in Figure 4i).

### 2.4 Tissue Adhesive and Lubricating Abilities of the Particles

**Figure 5:**
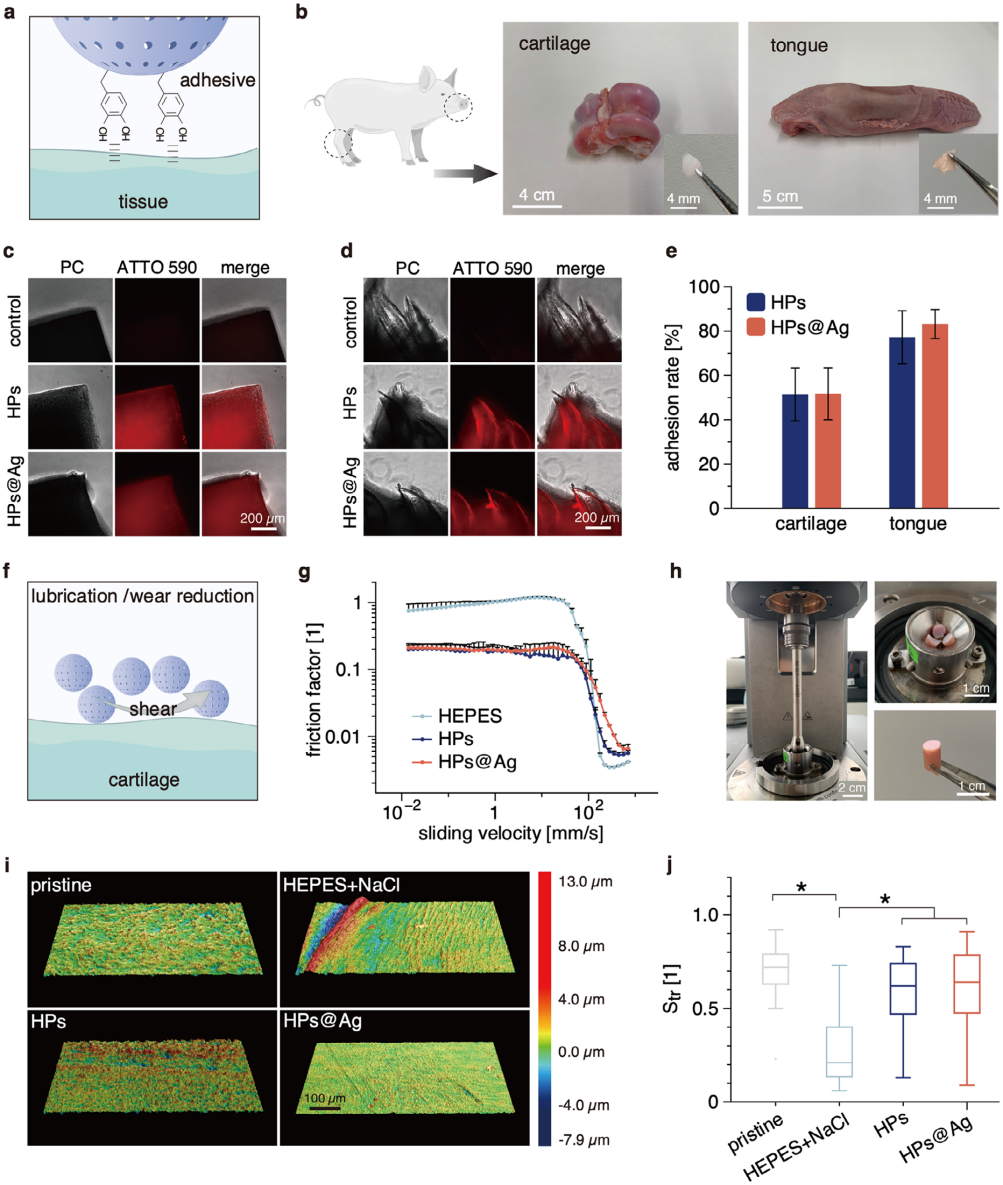
Tissue adhesion and lubricity of the HPs. a) Schematic illustration of the expected interactions between the catechol groups of the HPs and tissue surfaces. b) Images of porcine joint and tongue tissue samples used for the ex vivo tests. The insets show representative images of a cartilage piece and of a tissue piece from the dorsal surface of a porcine tongue as collected for testing the tissue‐adhesive abilities of the HPs. c,d) Phase contrast (PC) and fluorescence images of cartilage pieces and tongue tissue samples, respectively, after incubation with different HPs or HPs@Ag. The particles are covalently labeled with ATTO 590 (red signal). As controls, tissue samples are incubated with a buffer devoid of particles. e) Adhesion rates of the particles after incubation with the tissue samples for 2 h (*n*  =  5). f) Schematic illustration of the expected lubrication behavior of HPs. g) Friction curves obtained on PDMS when employing different solutions as lubricants; *n*  =  3. h) Tribological setup with osteochondral cylinders for assessing surface damage on cartilage tissue. Left: whole setup; upper right: sample holder with three cylindrical samples; lower right: osteochondral cylinders. i) 3D images of cartilage surfaces after tribological treatment with different lubricant solutions. The sample labeled as “pristine” represents the 3D image of a sample that was not subjected to tribological treatment. j) Texture aspect ratio (*S*_tr_) of the cartilage surfaces as calculated from 3D images (*n* ≥ 47). Data shown in (e,g) represents mean values; error bars denote the standard deviation. Asterisks (^*^) mark statistically significant differences determined in Mann–Whitney U tests based on a *p*‐value of 0.05.

In the last section of this study, we ask if the HPs and HPs@Ag possess tissue adhesive and lubricating abilities. This notion is based on previous findings reporting on the adhesive properties of PDA and the lubricating potential of mucins.^[^ ^83^, ^98^ ^]^ Since most drug delivery systems tend to suffer from a short retention time on cartilage tissue or the oral mucosa,^[^ ^99^, ^100^ ^]^ we test the tissue adhesive function of the particles on those two challenging targets, i.e., on porcine articular cartilage and the dorsal surface of the tongue (**Figure** **5**a,b). To do so, cartilage or tongue tissue pieces are incubated with the particles (which are covalently labeled with ATTO 590), washed to remove any unbound particles, and then visualized on a fluorescence microscope.

As shown in Figure 5c,d, we obtain clear fluorescence signals from such treated tissue samples for both HPs and HPs@Ag, confirming the tissue adhesive properties of both particle variants. We quantify this result by determining the adhesion rate for each particle type, i.e., by measuring the particle concentrations in the supernatant before and after incubation with those tissues. As summarized in Figure 5e, HPs and HPs@Ag exhibit similar adhesion rates on the two types of tissues, indicating that the addition of Ag^+^ “locks” does not compromise the adhesive function of the particles. Moreover, the obtained adhesion rates are higher on tongue tissue (≈80%) than on cartilage (≈50%), which could result from the rough surface structure of the tongue. We next ask which molecule(s) on the particles bring about the tissue adhesive properties of the particles. To examine this, PDA and mucin are labeled with fluorophores, and the corresponding solutions (i.e., pure PDA or pure mucin solutions) are incubated with the tissue samples. As shown in Figure S17 (Supporting Information), the fluorescence signals on the tissues indicate that not only PDA but also mucin can adhere to the cartilage or tongue samples, which can be attributed to the catechol and amine groups of PDA,^[^ ^83^, ^101^ ^]^ as well as to the long polymer chains (which lead to molecular entanglements) and the cationic termini of mucin.^[^ ^102^ ^,^ ^103^ ^]^

Next, the (putative) lubricating function of the particles (Figure 5f) is assessed using a rotational tribology setup employing a steel‐on‐PDMS pairing (which mimics a hard‐on‐soft interface, as, e.g., present in between the tongue and the palate). Since the particles bind well to challenging surfaces such as cartilage tissue and comprise well‐hydrated biomacromolecules, we do expect them to possess a lubricating potential, as the requirements for sacrificial layer formation and hydration lubrication are met. And indeed, as shown in Figure 5g, both HP and HP@Ag solutions reduce the friction coefficient in our setup compared to HEPES buffer. The friction factors obtained for the particle solutions are reduced by ≈80% in the boundary lubrication regime (which is the most challenging regime from a tribological point of view), and are still lower than those found for the HEPES group in the mixed lubrication regime, i.e., up to sliding speeds of 110 mm s^−1^ (which is well beyond the sliding speeds occurring in the human knee joints during walking).^[^ ^104^ ^]^ The good lubricity of the particles should mostly result from the mucin molecules, since mucin solutions strongly reduce friction (Figure S18, Supporting Information), whereas PDA solutions do not (which perform similarly to simple HEPES buffer, i.e., the control group).

Having observed a good lubricating function of the HPs, they could potentially be employed to treat early‐stage osteoarthritis, i.e., to prevent further wear formation on articular cartilage.^[^ ^105^ ^]^ Thus, we next examine the wear prevention capacities of the particles on porcine cartilage samples. To do so, we prepare osteochondral cylinders from the cartilage tissue of porcine joints and install them into the sample holder of the tribometer to obtain a steel‐on‐cartilage configuration (Figure 5h), similarly to what was described in Boettcher et al.^[^ ^106^ ^]^ Before and after tribological treatment, 3D topographical images of the cartilage surfaces are obtained using a laser scanning microscope. When using particle‐free HEPES buffer as a lubricant, clear wear tracks are observed on the surface of the cartilage samples, but those wear tracks are absent in pristine (untreated) cartilage samples (Figure 5i). In contrast, when we use one of the two particle solutions as a lubricant during tribological treatment, these wear tracks are much less pronounced. To quantify this impression, the texture aspect ratio (*S*_tr_) is calculated from the topographical images. The theoretically possible values of this metrological parameter range from 0 to 1; the closer *S*_tr_ is to 0, the less isotropic the surface morphology is, thus indicating strong surface alterations (= wear formation). As shown in Figure 5j, the *S*_tr_ values obtained for the HEPES group are significantly lower than the values obtained for the pristine samples, which confirms that this parameter is suitable for identifying the wear tracks we observe in the topographical images. Moreover, using the particle solutions as a lubricant, the calculated *S*_tr_ values are significantly higher than those obtained for the HEPES group and have median values close to those of the pristine (untreated) control group. Taken together, these results indeed nicely demonstrate the abilities of HPs and HPs@Ag to mitigate wear formation on cartilage surfaces.

## 3 Conclusion

In summary, PDA‐mucin blends are, for the first time, successfully fabricated into HPs with multifunctional properties. By employing different “locking” agents to crosslink the PDA‐mucin network, the permeability of the shells can be tuned to trap cargos inside the HPs. Among all the tested “locks”, we obtain the highest encapsulation efficiency for Ag^+^ ions, and this result agrees with a reduction in the pore size of the HP shells observed via SEM. Interestingly, such obtained HPs@Ag exhibit faster release kinetics when exposed to H_2_O_2_ or glucose. Furthermore, we demonstrate the ability of HPs and HPs@Ag to scavenge free radicals and show that, depending on the locking agent used, the HPs can either protect cells or promote cell death in an H_2_O_2_‐rich environment. Moreover, both HPs and HPs@Ag exhibit good tissue adhesion, lubricity, and wear prevention capabilities. As a potential application example, our HPs may be used for treating osteoarthritis,^[^ ^36^, ^99^ ^]^ as they can prolong drug retention, reduce the oxidative stress in the diseased tissue, lubricate the cartilage surface, and mitigate the formation of (further) surface damage.

In addition to demonstrating the broad range of functionalities provided by our PDA‐mucin HPs, our study also introduces a new approach for fabricating biopolymer‐based HPs with the aid of PDA. This approach should be applicable to many other biocompatible polymers such as alginate, dextrans, gelatin, and hyaluronic acid (HA).^[^ ^35^, ^107^, ^108^ ^]^ Then, by leveraging the distinct properties of different biopolymers, it might be possible to create HPs with other desirable functionalities and tailored responsive behavior. For example, HA macromolecules exhibit tumor‐targeting properties and are degradable in the tumor microenvironment;^[^ ^109^, ^110^ ^]^ thus, HA‐based HPs could be produced for tumor‐targeted drug delivery and locus‐specific drug release. Furthermore, future work may explore additional options for “locking” molecules such as Zn^2+^, Mn^2+^, and DNA strands. Such‐generated HPs, with the help of those “locks”, could be useful for applications in catalysis, imaging, or intracellular miRNA‐triggered drug release.^[^ ^20^, ^52^ ^]^ In addition, different conditions for dopamine oxidation can be employed for fabricating the HPs, which could lead to diverse particle structures and improved functionalities such as higher free radical scavenging efficiency.^[^ ^86^ ^]^ Overall, we anticipate that our PDA‐biopolymer‐based HPs will open new avenues for the development of multifunctional drug delivery systems.

## 4 Experimental Section

### Molecules

Calcium carbonate (CaCO_3_) microparticles (6 µm: PL‐CA6; 3 µm: PL‐CA3) were purchased from PlasmaChem GmbH (Berlin, Germany). Dopamine hydrochloride (H8502) and fluorescein isothiocyanate‐carboxymethyl‐dextrans (FITC‐CM‐dextran) with different molecular weights (4 kDa, 68059; 70 kDa, 53471; 150 kDa, 74817) were obtained from Sigma–Aldrich (Darmstadt, Germany). 4‐(2‐hydroxyethyl)‐1‐piperazineethanesulfonic acid (HEPES, HN78.1) and ethylenediamine tetraacetic acid (EDTA; 8040.2) were obtained from Carl Roth GmbH (Karlsruhe, Germany). 2,2‐diphenyl‐1‐picrylhydrazyl (DPPH•, 44150) and 2,2′‐azino‐bis(3‐ethylbenzothiazoline‐6‐sulfonic acid) diammonium salt (ABTS, J65535) were purchased from Thermo Scientific (Alfa Aesar, USA).

### Fabrication of DM HPs

The co‐deposition of dopamine and mucin molecules onto CaCO_3_ microparticles was achieved by a process similar to that described by Kang et al.^[^ ^43^ ^]^ In detail, a solution containing 8 mg mL^−1^ dopamine and 4 mg mL^−1^ lab‐purified mucin (MUC5AC, the purification steps were described by Marczynski et al.)^[^ ^111^ ^]^ was prepared in HEPES buffer (20 mm, pH 8.0), followed by mixing the dopamine/mucin solution with a CaCO_3_ suspension (500 mg mL^−1^ in ddH_2_O, which corresponds to 8.25 × 10^6^ particles mL^−1^) at a volume ratio of 1:1. The condition of pH 8.0 was chosen to allow for an efficient self‐polymerization of dopamine.^[^ ^112^ ^]^ The sample was incubated at 37 °C while gently shaking. The incubation time was 1.5 h unless specified otherwise. Subsequently, the particles were washed twice with ddH_2_O using centrifugation (2000 g, 2 min). The obtained CaCO_3_ microparticles carrying a DM coating were referred to as CaCO_3_@DM particles in this manuscript. To dissolve the CaCO_3_ cores, 1 mL of the CaCO_3_@DM particles (8.25 × 10^6^ particles mL^−1^) was mixed with 40 mL of an EDTA solution (0.4 m EDTA in ddH_2_O, pH 8.0) and shaken for 1 h at room temperature. Then, the obtained DM HPs were washed three times with ddH_2_O using centrifugation (2000 g, 2 min), and were resuspended in ddH_2_O for further use. The mass of CaCO_3_ used for fabrication, the corresponding mass of obtained HPs, and the corresponding obtained particle count are listed in Figure S7 (Supporting Information).

### Fluorescence Imaging of the Particles

Mucins were covalently labeled with the fluorophore Atto‐590 (AD 590, ATTO‐TEC GmbH, Germany) by employing carbodiimide coupling following the steps described in Fan et al.^[^ ^61^ ^]^ The obtained fluorescent mucins were used to fabricate CaCO_3_@DM particles and DM HPs for imaging. To do so, 10 µL of the particle solutions (i.e., CaCO_3_, CaCO_3_@DM, or DM HPs) at a concentration of 3.3 × 10^5^ particles mL^−1^ were added onto a glass slide (631‐1550, VWR) and covered with a cover slip (7695026, LABSOLUTE), followed by visualizing the particles on a DMi8 Leica microscope (Leica Microsystems, Wetzlar, Germany) in both, bright field and fluorescence mode (excitation/emission wavelength: 560/630 nm) using a 63× objective (HC PLFLUOTAR L 63x/0.7 dry, Leica).

### Assessing the Influence of the Co‐Incubation Time on the Yield Rate of DM HPs

To test the yield rate of the HPs, 300 µL of the CaCO_3_ solution was used for producing the DM HPs following the steps outlined above. Here, different co‐incubation times of CaCO_3_ with DM were used, i.e., 0.25, 0.5, 1.0, 1.5, 2, 3, or 4 h. The obtained DM HPs were then resuspended in 300 µL of ddH_2_O. The concentrations of the original CaCO_3_ microparticles and the created DM HPs were determined using a hemocytometer (0640730, Thoma) and by visualizing the samples on a DMi8 Leica microscope under phase contrast mode using a 63× objective. The respective yield rates of DM HPs were then calculated by normalizing the concentration of DM HPs to that of the CaCO_3_ microparticles.

### Absorbance Spectra of the Particles

For testing the absorbance behavior of the particles, 1 mL of each sample solution was added into a semi‐micro UV‐cuvette (759150, BRAND, Germany), and the corresponding absorbance spectrum was determined using a plate reader (SpectraMax ABS Plus, Molecular Devices, San Jose, USA) in a wavelength range from 200 to 350 nm (using increments of 2 nm). Here, ddH_2_O, a CaCO_3_ solution (3.3 × 10^4^ particles mL^−1^), a CaCO_3_@DM solution (3.3 × 10^4^ particles mL^−1^), a mucin solution (0.1 mg mL^−1^), a PDA solution (0.02 mg mL^−1^), and solutions of DM HPs at different concentrations (i.e., 2 × 10^5^, 1 × 10^5^, and 0.6 × 10^5^ particles mL^−1^) were tested. Since all samples were suspended in ddH_2_O, pure ddH_2_O was used as a blank control. The absorbance spectra were calculated by subtracting the absorbance values obtained for the blank control at each wavelength from the ones obtained for the samples.

### Zeta Potential of the Particles

The zeta potential values of the particles (i.e., CaCO_3_, CaCO_3_@DM, or DM HPs) were determined using a Litesizer 500 (Anton Paar, Austria) equipped with a 35 mW laser diode (λ  =  658 nm). For those measurements, the particles were suspended in 0.9 mL of 10 mm HEPES buffer (pH 7.4) at a concentration of 8000 particles mL^−1^. Then, the solutions were injected into a capillary cuvette (Omega cuvette, Anton Paar), which was inserted into the Litesizer device. The zeta potential of each sample was determined in automatic mode (T  =  25 °C, equilibration time: 30 s) using the Smoluchowski approximation of the Henry equation (Henry factor: 1.5).

### Size Distribution of the Particles

CaCO_3_ particles with sizes of either 6 or 3 µm (i.e., CaCO_3_ template I and CaCO_3_ template II, respectively) were used as templates to fabricate the corresponding CaCO_3_@DM and DM HPs following the steps outlined above. The obtained particle solutions (3.3 × 10^5^ particles mL^−1^ in ddH_2_O) were added onto a glass slide and covered with a cover slip. Then, bright field images of the microparticles were acquired on a DMi8 Leica microscope using a 63× lens. The diameters of the microparticles were measured by annotating their bright field images using the software ImageJ Fiji (version: 2.9.0/1.53t).

### Fabrication of HPs from Other Biopolymers

Instead of using mucins to produce HPs, two other biopolymers were chosen and co‐deposited onto the CaCO_3_ microparticles together with dopamine. These two polymers were poly‐D‐lysine (PDL; AB349570, abcr GmbH) and dextran (FITC‐labeled; 46946, Sigma–Aldrich). PDL molecules were covalently labeled with ATTO 488 carboxy (AD 488‐25, ATTO‐TEC GmbH) by employing carbodiimide coupling following the steps described in Fan et al.^[^ ^61^ ^]^ Then, either PDL or dextran solutions were mixed with a dopamine solution, and the mixtures were incubated with CaCO_3_ solutions to obtain final concentrations of the biopolymer, dopamine, and CaCO_3_ of 2, 4, and 10 mg mL^−1^, respectively. After 1.5 h of incubation at 37 °C during gentle shaking, the particles were washed twice with ddH_2_O using centrifugation (2000 g, 2 min) and were resuspended in ddH_2_O at a concentration of 3.3 × 10^5^ particles mL^−1^. Then, 4 µL of each particle solution was mixed with 8 µL of EDTA (0.4 m, pH 8.0). This mixture was added onto a glass slide, covered with a cover slip, and sealed with vacuum grease (HM‐4198, Humboldt Mfg. Co.). After an incubation time of 0.5 h, the obtained HPs were visualized on a DMi8 Leica microscope in both, bright field and fluorescence mode (excitation/emission wavelength: 495/519 nm) using a 63× objective.

### Drug Loading into HPs

FITC‐CM‐dextrans with three different molecular weights (4k, 70k, 150k) were used as model cargos for drug loading tests. To load these molecules into HPs, 10 µL of HPs (8.25 × 10^6^ particles mL^−1^ in ddH_2_O) was mixed with 10 µL of a FITC‐CM‐dextran solution (4 mg mL^−1^ in HEPES, pH 7.0), and then incubated for 30 min at room temperature. Subsequently, 20 µL of a crosslinker (“lock”) solution was added to the mixture and incubated at room temperature for 1 min; to obtain a control group without any crosslinkers, 20 µL of ddH_2_O was added to the dextran/HP mixture. The crosslinkers tested here include lectin (0.2 mg mL^−1^ in ddH_2_O, from *Triticum vulgaris*; L9640, Sigma–Aldrich), CaCl_2_ (20 mm in HEPES, pH 7.0; CN93.1, Carl Roth), PDL (0.25 mg mL^−1^ in HEPES, pH 7.0), and AgNO_3_ (40 mm in ddH_2_O; 2246.1, Carl Roth). After the crosslinking step, the dextran‐loaded HPs were washed with ddH_2_O for three times using centrifugation (2000 g, 2 min) and were resuspended in ddH_2_O for imaging under a DMi8 Leica microscope in both, phase contrast and fluorescence mode (excitation/emission wavelength: 495/519 nm) using a 63× objective.

For determining the encapsulation efficiency of the different dextrans into the HPs, 10 µL of the HP solution was first mixed with 10 µL of one of the dextran solutions, and then with 20 µL of one of the crosslinker solutions, following the same steps described above. Then, 400 µL of ddH_2_O was added to the mixture, followed by centrifugation (2000 g, 2 min), and 300 µL of supernatant was removed for testing its fluorescence intensity (FI). Afterward, each centrifuged particle solution was thoroughly mixed with 600 µL of ddH_2_O, centrifuged again, followed by removing 300 µL of the supernatant for testing the FI. Subsequently, each (second‐time) centrifuged particle solution was thoroughly mixed with 900 µL of ddH_2_O, centrifuged again, followed by testing the FI of the supernatants. Those three consecutive centrifugation steps were conducted to ensure that all unbound (or weakly bound) cargo molecules were collected (as the FI of the supernatant was close to zero after the third centrifugation step). As blank controls, 10 µL of ddH_2_O (instead of the HP solutions) was first mixed with 10 µL of the dextrans and then with 20 µL of one of the crosslinkers, following the steps described above. Then, 400 µL of ddH_2_O was added to each solution, followed by testing the FI. To do so, the corresponding supernatants were transferred into a 96‐well plate (black, costar; 100 µL per well), and the fluorescence intensity of the samples was determined using a plate reader (Varioskan LUX, Thermo Fisher) at an excitation/emission wavelength of 495/520 nm. The encapsulation efficiency of HPs was then calculated based on the FI and the volume of the solutions after each washing step according to the following equation:

(1) $$\begin{array}{ccc} & & encapsulation\;efficiency\;\;\left[\%\right]\\ \; & & =\left(\frac{amount\;of\;loaded\;dextrans\;\left[mg\right]}{amount\;of\;initial\;dextran\;feed\;\left[mg\right]}\right)\times 100\\ \; & & =\left(1-\frac{FI_{1}\times 300+FI_{2}\times 300+FI_{3}\times 1340}{FI_{blank}\times 440}\right)\times 100 \end{array}$$

where FI_i_ is the FI of the supernatant of the HPs after the i‐th centrifugation step, and FI_blank_ is the FI of the blank‐control solutions for the corresponding “locks”.

### SEM Imaging

The particles were examined using a scanning electron microscope (SEM, Jeol JSM‐7600F, Jeol GmbH, Germany). Except for CaCO_3_, all samples were sputtered with gold. Before sputtering, the particles (suspended in ddH_2_O) were dried by evaporating the water under vacuum (for the CaCO_3_@DM and the HPs) or by heating the samples to vaporize the water (for HPs@Ag). Similar morphology and porosity of the HPs@Ag were obtained when using vacuum drying and heat drying; however, to avoid background artifacts, heat drying was employed for preparing the samples of HPs@Ag. Imaging was conducted at a working distance of 10 mm. Owing to charging effects, the acceleration voltage was varied between 1 and 15 kV, depending on the sample.

### Drug Release from HPs

In these tests, fluorescently labeled CM‐dextran served as a model cargo. The HPs were first loaded with FITC‐CM‐dextran (4 kDa) and crosslinked with Ag^+^ following the steps described above. To test the dextran release profile of HPs under different conditions, 25 µL of the dextran‐loaded HPs (8.25 × 10^6^ particles mL^−1^ in ddH_2_O) was added to 300 µL of either ddH_2_O, D‐PBS (pH 7.4), D‐PBS enriched with 10 mm H_2_O_2_ (pH 7.4), or D‐PBS enriched with 8 mg mL^−1^ glucose (pH 7.4). Subsequently, the HPs were incubated in these different solutions at 37 °C while gently shaking for different time spans (10 min, 3 h, 23.5 h, 44 h, 68 h, 92 h). To test the amount of released dextrans, the HP solutions were centrifuged (2000 g, 2 min), and 100 µL of the supernatant from each sample was transferred to a black 96‐well plate. Then, 100 µL of D‐PBS (pH 7.4) was added to each well (as FITC exhibits lower fluorescence intensity in ddH_2_O). The fluorescence intensity of each sample was determined using a plate reader at an excitation/emission wavelength of 495/520 nm. Every time after removing the supernatant, 100 µL of the corresponding fresh buffer was added to the HP solutions to replace the supernatant. To calculate the amount of released dextrans, standard curves of the FITC‐CM‐dextran were also obtained for each buffer condition and at each time point to take any putative influence of the buffer conditions into account and to avoid fading of the fluorescence in the solutions, respectively (see Figure S19, Supporting Information for the calibration curves). The percentage of the released dextran amount was finally calculated by normalizing the released amount of dextrans to the total dextran amount.

### Free Radical Scavenging

Two assays were employed to test the free radical scavenging capability of the particles, i.e., DPPH• and ABTS•^+^ assays, following the steps reported by Lu et al.^[^ ^113^ ^]^ In the DPPH• assay, 40 µL of each HP sample (prepared in ddH_2_O) was mixed with 360 µL of DPPH• (55.5 µm, prepared in ethanol) to obtain different final particle concentrations (0, 1.56, 3.12, 6.25, 12.5, or 25 µg mL^−1^); then, the mixed samples were vigorously shaken for 30 min in the dark. Subsequently, the mixtures were centrifuged (2000 g, 5 min) and the corresponding supernatants were transferred to a 96‐well plate (100 µL well^−1^). The sample absorbance at a wavelength of 517 nm was determined using a plate reader. The DPPH• scavenging percentage was calculated according to the following formula:

(2) $$DPPH•scavenging\left[\%\right]=\left(1-\frac{A-A_{0}}{A_{1}-A_{0}}\right)\times 100$$

where A_0_ is the absorbance of ethanol, A_1_ is the absorbance of the reaction system without particles, and A is the absorbance of the reaction system with particles.

In the ABTS•^+^ assay, the ABTS solution (prepared in ddH_2_O) was mixed with a potassium persulfate solution (prepared in ddH_2_O) to obtain final concentrations of 3.7 and 1.3 mm, respectively; then, the mixture was allowed to react in the dark for 18 h to generate ABTS•^+^. Afterward, 8 µL of the generated ABTS•^+^ solution was mixed with 492 µL of each sample solution to obtain different particle concentrations (0, 1.56, 3.12, 6.25, 12.5, or 25 µg mL^−1^). After shaking for 6 min, the mixtures were centrifuged (2000g, 5 min) and the corresponding supernatants were transferred to a 96‐well plate (100 µL well^−1^). The sample absorbance at a wavelength of 734 nm was determined using a plate reader. The ABTS•^+^ scavenging percentage was then calculated according to the following formula:

(3) $$ABTS{•}^{+}scavenging\left[\%\right]=\left(1-\frac{A-A_{0}}{A_{1}-A_{0}}\right)\times 100$$

where A_0_ is the absorbance of ddH_2_O, A_1_ is the absorbance of the reaction system without particles, and A is the absorbance of the reaction system with particles.

### Cell Tests

Human epithelial cells (HeLa) were cultured in minimum essential medium (MEM; Sigma–Aldrich) containing 10% (v/v) fetal bovine serum (FBS; Sigma–Aldrich), 2 mm L‐glutamine (Sigma–Aldrich), 1% (v/v) of a non‐essential amino acid solution (Sigma–Aldrich), and 100 U mL^−1^ penicillin‐streptomycin (Sigma–Aldrich). The cells were maintained at 37 °C in a humidified atmosphere with 5% CO_2_.

To test the ROS levels in the cells, 2′,7′‐dichlorodihydrofluorescein diacetate (DCFH‐DA; HY‐D0940, MCE) was used to stain the cells. To do so, the HeLa cells were seeded into a 96‐well plate (5000 cells well^−1^) and incubated overnight. Afterward, the cells were incubated with DCFH‐DA (10 µM in D‐PBS) for 0.5 h, and then washed twice with D‐PBS (containing 0.9 mm CaCl_2_ and 0.5 mm MgCl_2_). Subsequently, the cells were incubated with a medium containing hydrogen peroxide (H_2_O_2_; 0 or 400 µm) and particles (0 or 100 µg mL^−1^). After 55 min, the cells were imaged on a DMi8 Leica microscope in both phase contrast and fluorescence mode (excitation/emission wavelength: 495/519 nm) using a 10× objective (N PLAN 10×/0.25 DRY). The fluorescence intensity of each image was quantified using Leica software (LAS X, version: 3.0.4.16529).

To test the protective/toxic effects of the particles on the cells in the presence of H_2_O_2_, cell culture media containing H_2_O_2_ (0 or 10 mm) and particles (0 or 50 µg mL^−1^) were incubated with HeLa cells at 37 °C for 5 h. Subsequently, those solutions were replaced with Live/Dead staining solutions (Invitrogen, L3224), and the cells were incubated for 30 min. After replacing the staining solutions with D‐PBS, the cells were imaged on a DMi8 Leica microscope in both phase contrast and fluorescence mode (excitation/emission wavelength: 495/519 nm, and 560/630 nm) using a 10× objective (N PLAN 10×/0.25 DRY). The numbers of live and dead cells in each image were counted, and the percentage of live cells was calculated by normalizing the number of live cells to the total number of cells obtained for each image.

### Ex Vivo Particle Adhesion Tests to Tissues

Porcine cartilages were freshly obtained from Metzgerei Boneberger GmbH (Neufahrn, Germany), and porcine tongue tissue was obtained from Alber “Der Metzger” GmbH (Marktl am Inn, Germany). To test the tissue‐adhesive function of the particles, small pieces of cartilage (1.5 mm × 1.5 mm × 0.6 mm) or tongue samples (1.5 mm × 1.5 mm × 0.3 mm) were incubated with particle solutions (50 µg mL^−1^ prepared in MEM) at 37 °C while gently shaking. After 2 h, the tissues were washed with MEM and then immersed in fresh MEM for imaging on a DMi8 Leica microscope in both phase contrast and fluorescence mode (excitation/emission wavelength: 560/630 nm) using a 20× objective (HC PL FLUOTAR L 20×/0.40 DRY). All images were acquired at the same light intensity and exposure time. To test the adhesion rate of the particles, tissue pieces were cut into a round shape and transferred to a 96‐well plate to fully cover the bottom of the wells. Here, each tissue piece was immersed in 100 µL of a particle solution (50 µg mL^−1^ in MEM) for 2 h while gently shaking at 37 °C. The particle concentrations in these solutions before and after incubation with the tissue pieces were determined using a hemocytometer (0640730, Thoma) under a DMi8 Leica microscope. The adhesion rate was then calculated according to the following equation:

(4) $$\begin{array}{ccc} & & adhesion\;rate\;\left[\%\right]\\ & & =\left(1-\frac{particle\;concentration\;after\;the\;incubation\;\left[counts/mL\right]}{particle\;concentration\;before\;the\;incubation\;\left[counts/mL\right]}\right)\times 100 \end{array}$$

### Rotational Tribology

Friction measurements were performed using a commercial shear rheometer (MCR 302, Anton Paar, Graz, Austria) equipped with a tribology unit (T‐PTD 200, Anton Paar) and a ball‐on‐3‐cylinders geometry. The friction partners were a 12.7‐diameter steel ball (1.4301, *Sq* <0.2 µm, Kugel Pompel, Vienna, Austria) and three cylindrical PDMS pins (*d*  =  7 mm). The PDMS cylinders were produced by mixing PDMS prepolymer and curing agent (Sylgard 184, Dow Corning, Midland, MI, USA) in a ratio of 10:1. A vacuum pump was used for a minimum of 1 h to remove bubbles from the mixture; afterward, the PDMS was poured into a custom‐made steel mold and cured at 80 °C overnight. The PDMS pins were then removed from the mold and washed with 80% ethanol and DI water prior to use.

Friction measurements were conducted at a constant normal force of *F*_N_ =  6 N. The geometry of our setup resulted in a normal force of 2.8 N per pin. The contact pressure between steel and PDMS was estimated based on the Hertzian contact theory using the following formula:

(5)

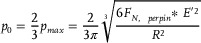

with:

(6) $$\frac{1}{E^{\prime }}=\frac{1-v_{1}^{2}}{E_{1}}+\frac{1-v_{2}^{2}}{E_{2}}$$

With typical material parameters for steel (*E*_1_ = 210 GPa, ν_1_ ≈ 0.30) and PDMS (*E*_2_ ≈ 2 MPa, ν_2_ ≈ 0.49), this corresponds to a normal pressure of ≈0.3 MPa. The sliding speed was changed from 700 to 0.001 mm s^−1^ with a measuring point duration of 10 s for each point (total number of acquired data points per curve = 48). For each measurement, 500 µL of a particle solution (8.25 × 10^6^ particles mL^−1^ in 20 mm HEPES, pH 7.0) was used; HEPES (20 mm, pH 7.0) buffer served as a control. Of note, the particle concentration of 8.25 × 10^6^ particles mL^−1^ was equivalent to 1 mg mL^−1^ of the HPs.

### Surface Damage Analysis on Tribologically Treated Osteochondral Cylinders

Osteochondral tissue was harvested from pig knee joints, which were obtained with closed articular capsules from Metzgerei Boneberger GmbH (Neufahrn, Germany). Osteochondral cylinders with a diameter of 5.5 mm were drilled out of the tissue as described in Boettcher et al.^[^ ^106^ ^]^ Prior to tribological treatment, the samples were immersed in 20 mm HEPES buffer (pH 7.0) supplemented with 154 mm NaCl (which will be referred to as HEPES+NaCl buffer in the main text) for at least 1 h to ensure an identical hydration state for all cartilage samples. Subsequently, a tribological treatment of the cartilage samples was conducted by employing a shear rheometer equipped with a tribology unit and the ball‐on‐3‐cylinders geometry mentioned above (section “*Rotational Tribology*”). In detail, the osteochondral cylinders were mounted into the sample holder and secured from the bottom and the side with screws; then, the holder was filled with 600 µL of a lubricant solution. The tested lubricant solutions include HEPES+NaCl buffer, as well as particle solutions comprising either 1 mg mL^−1^ HPs or 1 mg mL^−1^ HPs@Ag in HEPES+NaCl buffer. A steel sphere (Kugel Pompel, Austria, 1.4404, *d* = 12.7 mm, surface roughness *S*_q_ <0.2 µm) was rotated on top of the osteochondral cylinders using a normal force of 6 N and a sliding speed of 0.1 mm s^−1^ for a total duration of 3 h. After this treatment, the cartilage samples were carefully washed to remove residual lubricants, and then dried for 10 min to eliminate most water residues from the surface. Then, the sample surfaces were imaged on a laser scanning microscope (VK‐X1100 Keyence Corporation, Japan) equipped with a 20× objective. The software MultiFileAnalyzer (Keyence Corporation) was used to process and analyze the obtained 3D topographical images: the images were first corrected to remove the intrinsic curvature of the cartilage surface, followed by extracting the texture aspect ratio (*S*_tr_; for a definition of this parameter, please refer to ISO 25178) from each image.

### Statistical Analysis

All experimental data were expressed as mean ± standard deviation. Prior to each statistical analysis, the normal distribution of the measured data points was tested with a Shapiro–Wilk test. Homogeneity of variances was assessed with an F‐test. A Student's *t*‐test was performed for normally distributed populations with homogeneous variances, whereas a Welch's *t*‐test was used in the case of unequal variances. For non‐normal distributions, a Mann–Whitney U test was used. To detect statistical differences between more than two groups, one‐way Analysis of Variances (ANOVA) was performed. Excel with two add‐ins (i.e., Analysis ToolPak, and Xrealstats) was used to conduct all the statistical tests. The level for significance was always set to *p* <0.05, and significant differences were marked with an asterisk in the respective graphs; non‐significant differences were indicated with the abbreviation “n.s.” where applicable.

## Conflict of Interest

The authors declare no conflict of interest.

## Author Contributions

D.F. and O.L. designed the study. C.G. conducted the tribological experiments. Y.W. developed the protocol for fabricating the HPs and performed pre‐experiments. L.R. conducted SEM imaging and P.B. conducted FTIR measurements under the supervision of J.T. All other experiments were carried out by D.F. The manuscript was written by D.F. and O.L. and was critically revised by all authors.

## Supporting information



Supporting Information

## Data Availability

The data that support the findings of this study are available from the corresponding author upon reasonable request.
