# Supplementary material for: Multi‐Functional Polydopamine‐Mucin Hollow Particles Provide Tunable Shell Permeability, ROS Scavenging, Tissue Adhesion, and Lubricity for Biomedical Applications
Source: Small. 2025 Jul 4;21(34):2503238. doi: 10.1002/smll.202503238 (PMC12393020; doi:10.1002/smll.202503238)
Supplement: Supplementary file 1 — Supporting Information [file SMLL-21-2503238-s001.docx]

| Supporting Information |
| --- |
| **Multi-Functional Polydopamine-Mucin Hollow Particles Provide Tunable Shell Permeability, ROS Scavenging, Tissue Adhesion, and Lubricity for Biomedical Applications**  *Di Fan, Chiara Gunnella, Yukun Wang, Luca Reichert,* *Pedro Henrique da Rosa Braun, Jan Torgersen, and Oliver Lieleg** |
| D. Fan, C. Gunnella, Y. Wang, O. Lieleg  Department of Materials Engineering, School of Engineering and Design  Center for Protein Assemblies and Munich Institute of Biomedical Engineering  Technical University of Munich, Ernst-Otto-Fischer Str. 8, 85748 Garching, Germany  Email: [oliver.lieleg@tum.de](mailto:oliver.lieleg@tum.de)  L. Reichert, P. Braun, J. Torgersen  Department of Materials Engineering, School of Engineering and Design,  Institute of Materials Science,  Technical University of Munich, Boltzmannstraße 15, 85748 Garching, Germany  Keywords:  drug encapsulation, free radical scavenging, tissue adhesive, lubrication, wear prevention  *Corresponding author  ORCID: DF: 0000-0001-7291-2592; CG: 0009-0008-1135-963X; YW: 0009-0000-1967-8098; LR: 0009-0002-6266-8682; PB: 0000-0003-1488-206X; JT: 0000-0003-1675-8759; OL: 0000-0002-6874-7456. |

**1. Possible interactions between polydopamine (PDA) and mucin, and their molecular structures**

Under alkaline conditions, dopamine self-polymerizes into PDA (**Figure S1a**),^[1]^ which can bind to mucin glycoproteins (**Figure S1b**).^[2]^ The possible interactions between PDA and mucin include i) covalent links formed through Michael addition and Schiff-base reaction (where the quinone moieties on PDA react with the amine and thiol groups on mucin), and ii) noncovalent bonds such as cation-π interactions, electrostatic forces, hydrogen bonds, and hydrophobic forces.^[3,4]^

| 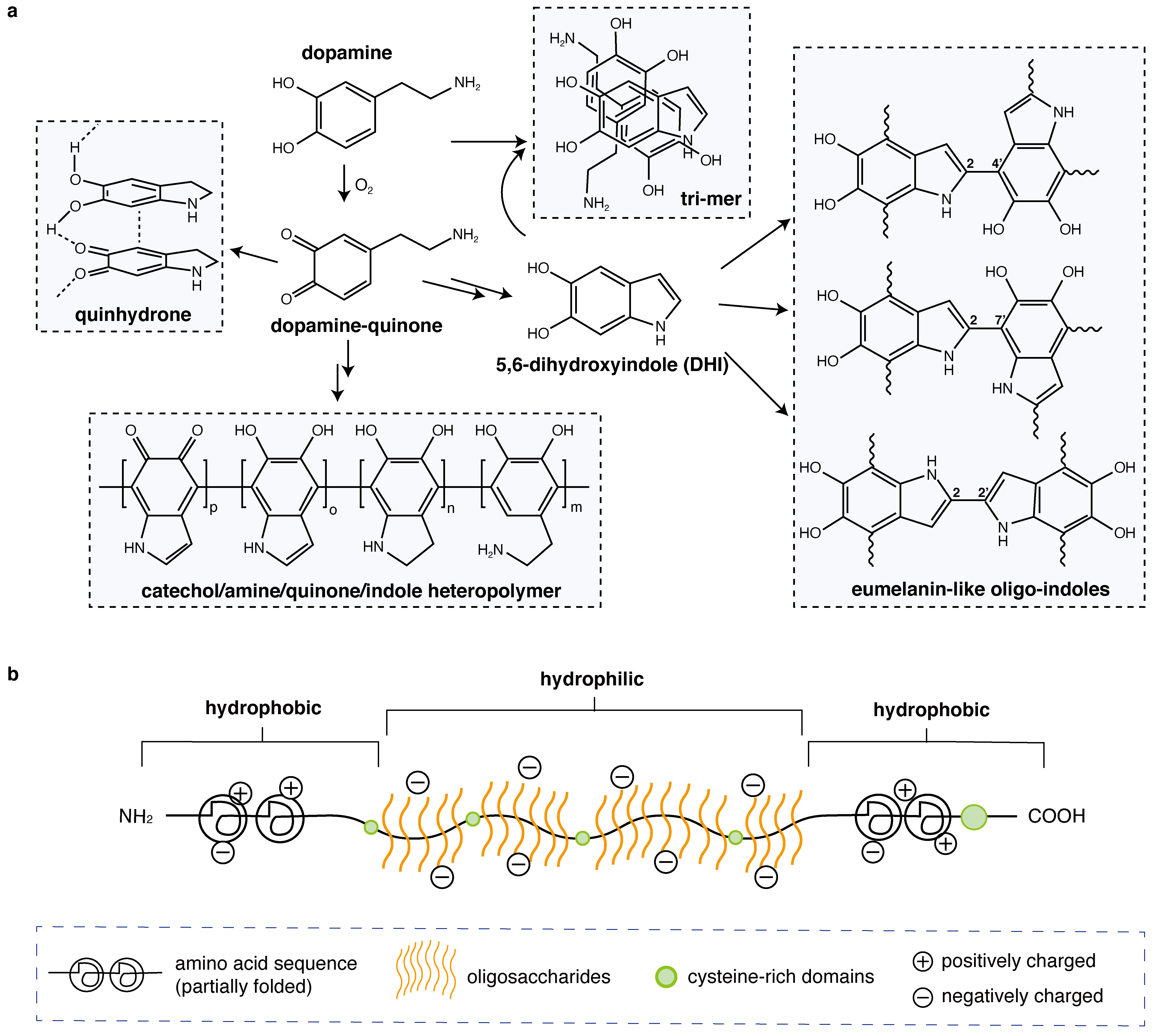 |
| --- |
| **Figure S1.** Molecular structures. (a) Dopamine, and possible formation pathways of different PDA variants (shown in the dashed boxes). (b) Mucin (MUC5AC). |

**2. Particle pellets obtained after centrifugation**

Calcium carbonate (CaCO_3_) microparticles (average size: ~6 µm) and PDA-mucin coated CaCO_3_ (CaCO_3_@DM) microparticles were suspended in double-distilled water (ddH_2_O) at a concentration of 3.3 × 10^5^ particles/mL. The images shown below were taken after centrifugation (2000 g, 2 min) of the solutions.

| 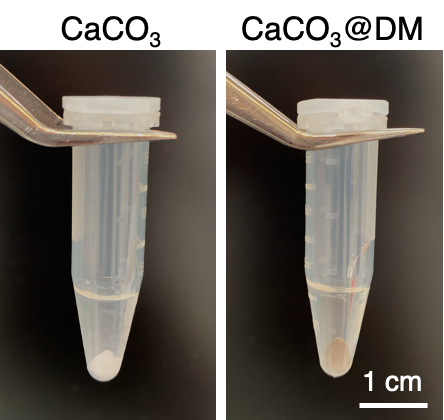 |
| --- |
| **Figure S2.** Images of the particle pellets obtained after centrifugation in ddH_2_O. The scale bar applies to both images. |

**3. Verifying the binding between PDA and mucin**

To verify the binding between PDA and mucin, we conducted quartz crystal microbalance with dissipation monitoring (QCM-D) measurements using a qcell T-Q2 platform (3T-Analytik, Germany) and gold chips as described previously.^[5]^ As shown in **Figure S3**, flushing a dopamine solution (4 mg/mL) across the crystal leads to a drop in the change of resonance frequency (Δf), indicating the adsorption of PDA to the surface. After a washing step, flowing a mucin solution (0.1 mg/mL) over the PDA-coated crystal results in a strong decrease in Δf, thus verifying the efficient binding between PDA and mucin molecules.

| 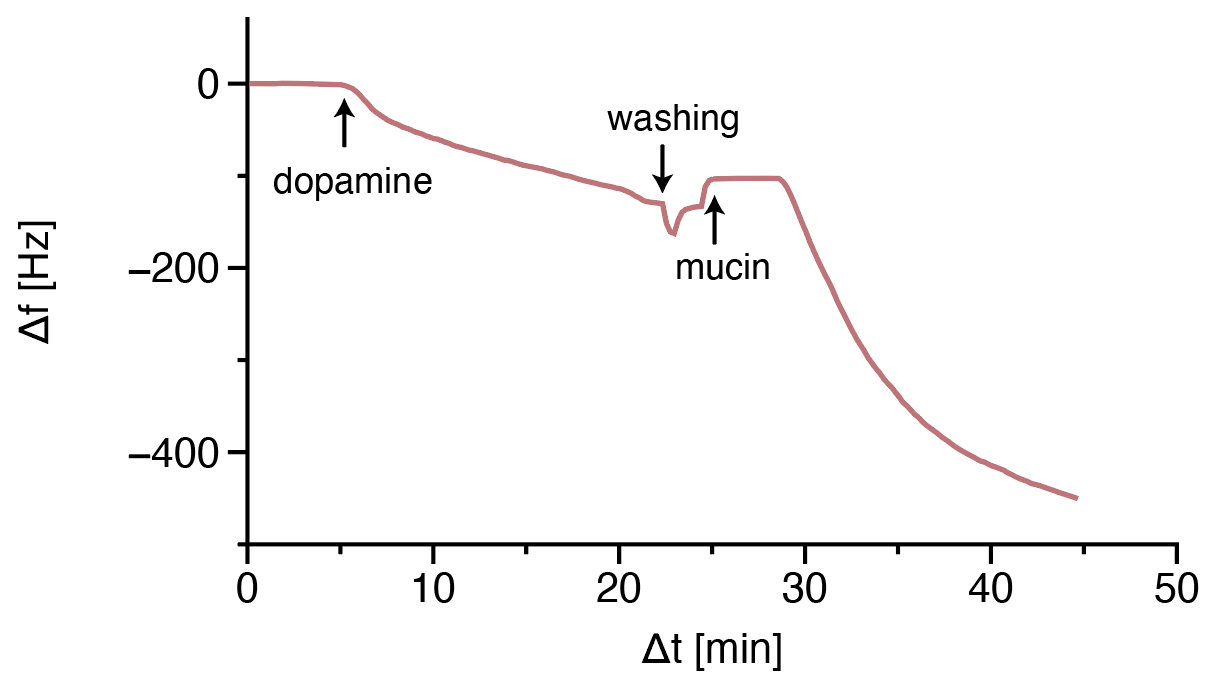 |
| --- |
| **Figure S3.** QCM-D measurements reporting on the binding between PDA and mucin. The arrows indicate the time points when different solutions were injected at different flow rates: a dopamine solution at 100 µL/min, HEPES buffer (20 mM, pH 7.0) at 1000 µL/min, and a mucin solution at 100 µL/min. |

**4. Zoomed-out images of the particles at each fabrication step**

As shown in **Figure S4**, zoom-out images of CaCO_3_, CaCO_3_@DM, and HPs were obtained following the steps described in the methods section “*Fluorescence imaging of the particles*” in the main text. Their corresponding zoomed-in images are shown in **Figure 2b** in the main text.

| 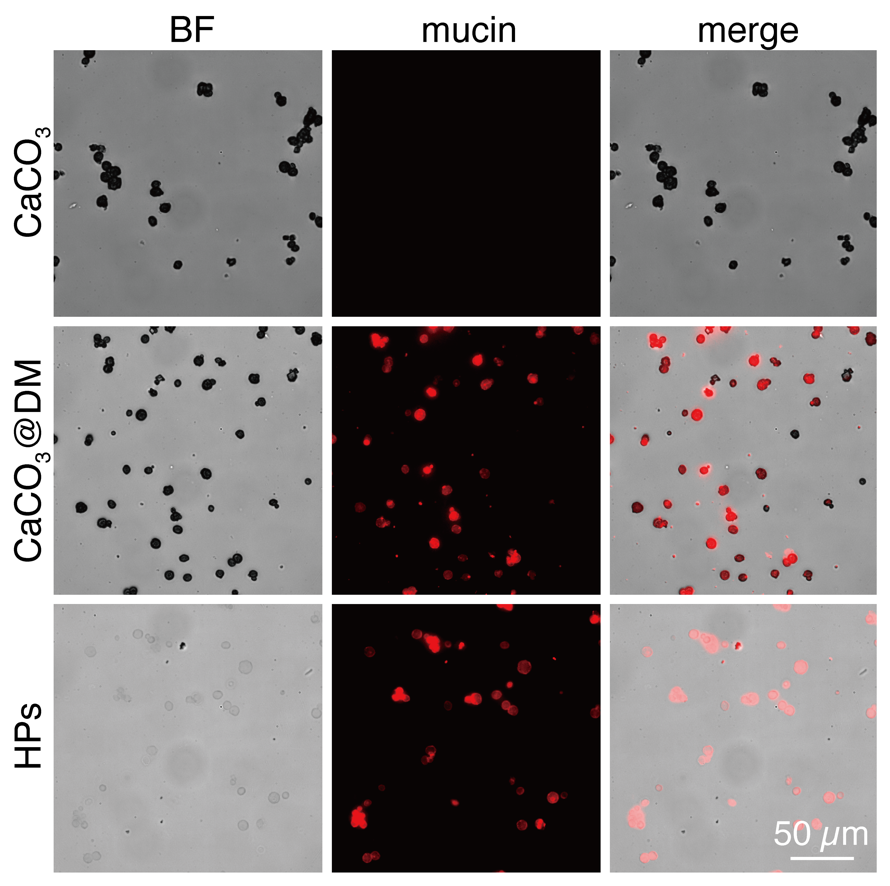 |
| --- |
| **Figure S4.** Bright field (BF) and fluorescence images of the particles in ddH_2_O. Mucins are covalently labelled with ATTO 590 (red signal). The scale bar applies to all images in this figure. |

**5. Dissolving process of the CaCO_3_ cores of CaCO_3_@DM in EDTA**

To visualize the dissolution of the CaCO_3_ cores upon contact with EDTA, time-lapse images of a CaCO_3_@DM sample were obtained. In detail, CaCO_3_@DMs were fabricated following the steps described in the section “Fabrication of DM HPs” of the manuscript. Afterward, 10 µL of a CaCO_3_@DM solution (prepared in ddH_2_O) was added to a glass slide and covered with a cover slip. The sample was mounted onto a DMi8 Leica microscope and focused on using a 63× lens. Then, an EDTA solution (0.4 M, pH 8.0) was added to the sample from the side of the cover slip and images were acquired in bright field mode. The results are shown in **Figure S5**.

| 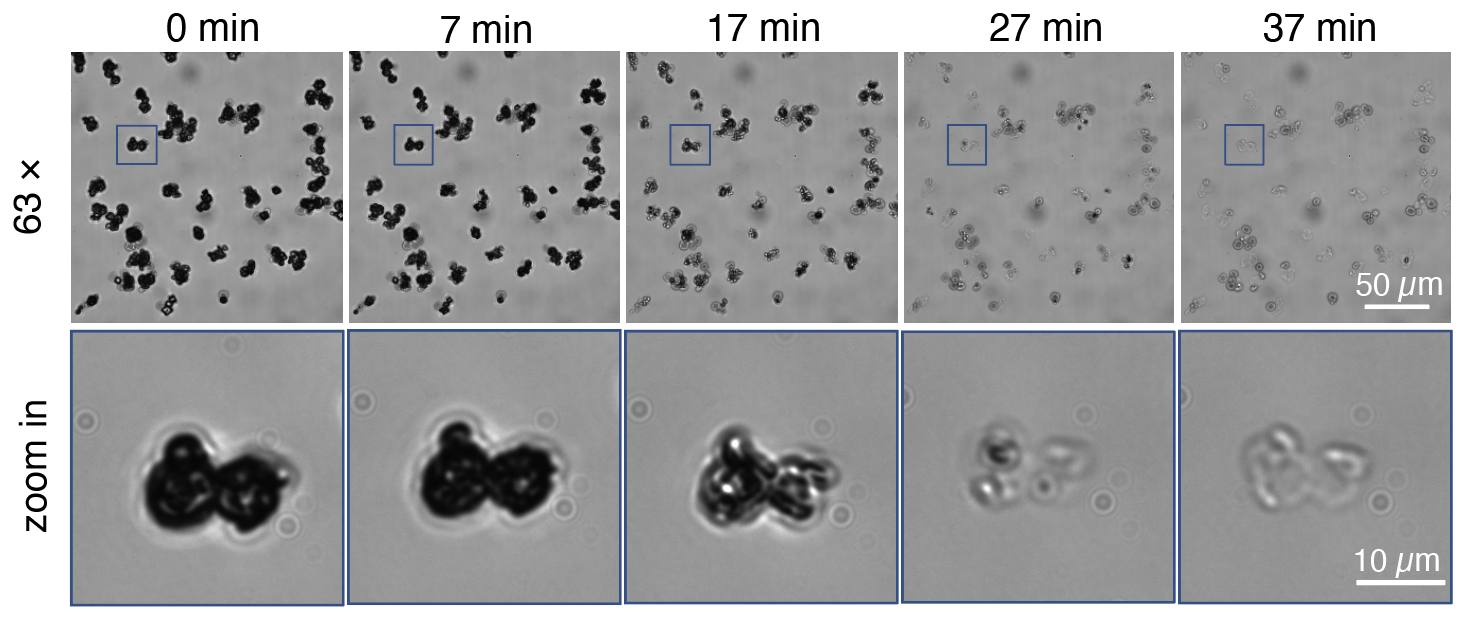 |
| --- |
| **Figure S5.** Dissolution of the CaCO_3_ cores of CaCO_3_@DM over time. The areas boxed in blue in the images from the upper row are shown at higher magnification in the bottom row. The scale bars apply to all images in the respective row. |

**6. Thickness of the particle shells**

The thickness of the particle shells was obtained by annotating the images of the particles and analyzing them using ImageJ Fiji. The HPs were prepared by employing different incubation time spans for the co-deposition step of PDA and mucin onto CaCO_3_ particles. The images were collected following the steps described in the methods part “*Assessing the influence of the co-incubation time on the yield rate of DM HPs*” of the main text. The obtained data is shown in **Figure S6**.

| 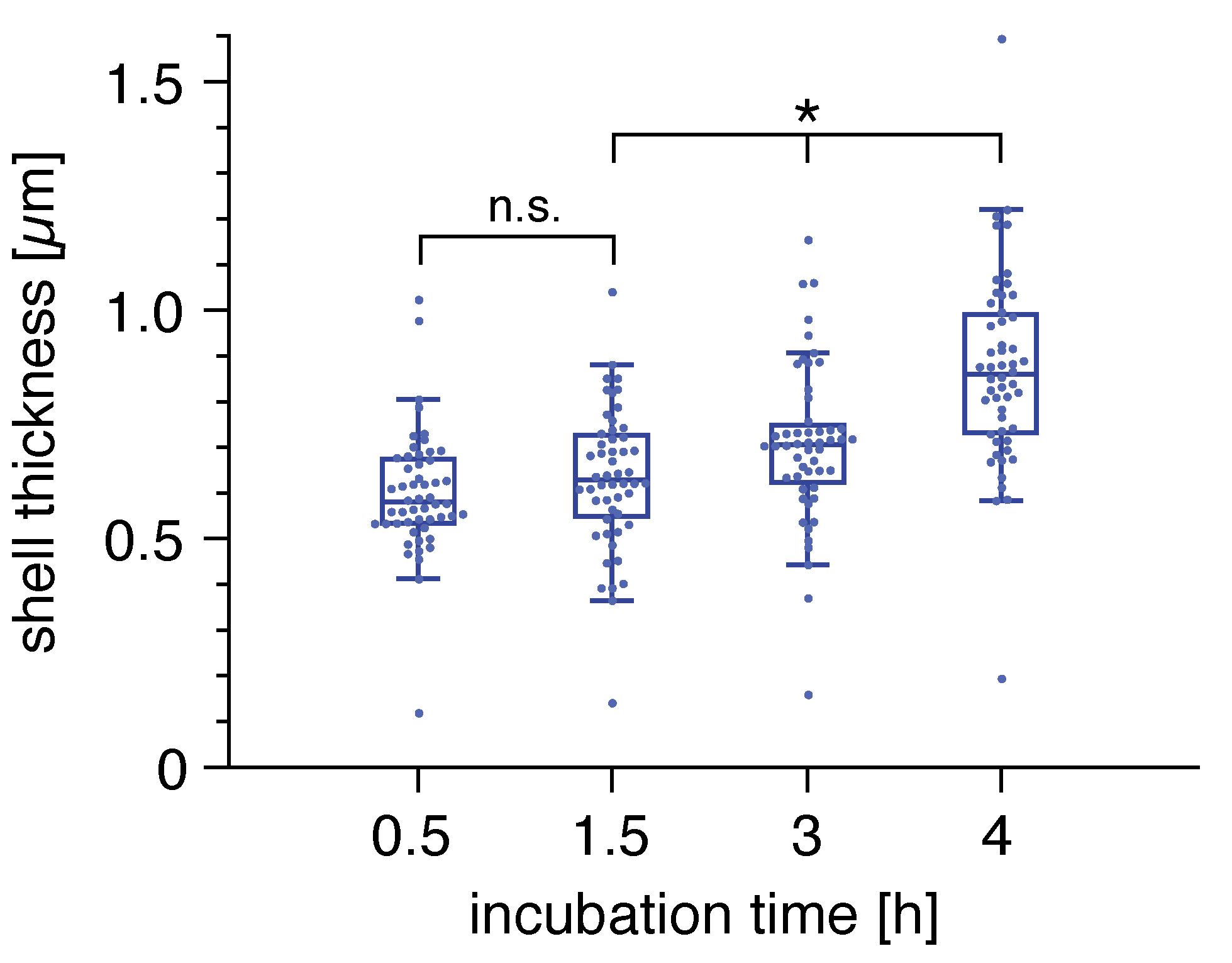 |
| --- |
| **Figure S6.** Thickness distributions of the HP shells when using different incubation time spans in the step of coating CaCO_3_ with PDA and mucin. *n* = 50. Asterisks (*) mark statistically significant differences determined in Mann-Whitney U tests based on a *p*-value of 0.05; “n.s.” denotes non-significant differences. |

**7. Density of the particles and mucin amount in each particle**

The particles were prepared following the steps described in the methods section “*Fabrication of DM HPs*” of the main text. Afterward, the diameter of HPs was obtained by annotating the images of HPs and analyzing them using ImageJ Fiji. Then, the volume of the annotated particles was calculated. The mass of the particles was obtained by lyophilization of the HP solutions and then weighing the HP powders. The particle count in the HP solutions was quantified using a hemocytometer (Thoma), which was mounted on a DMi8 Leica microscope; HP samples were examined under phase contrast mode using a 20× objective. With the measured mass of the HP powders and the obtained particle count, the average mass of the particles was calculated (*i.e.*, 0.1212 ng). The distribution of the particle density was then obtained by dividing the average mass of the particles by the volume of each particle (**Figure S7a**).

The amount of the materials used/produced during the fabrication process is summarized in **Figure S7b**. To quantify the average mass ratio of mucin in the particles, ATTO-594 labelled mucin was used for preparing the HPs. Then, the obtain HP solution (62.5 µg/mL in D-PBS, pH 7.4) was added to the wells of a 96-well plate (with black side walls; 100 µL per well), and the fluorescence intensity of the samples was obtained using a plate reader (Varioskan LUX, Thermo Fisher) at an excitation/emission wavelength of 601/626 nm. The mucin concentration in the HP sample was then calculated based on a standard curve of pure mucin-ATTO-594 solutions which relates the fluorescence intensity to the mucin concentration.

| 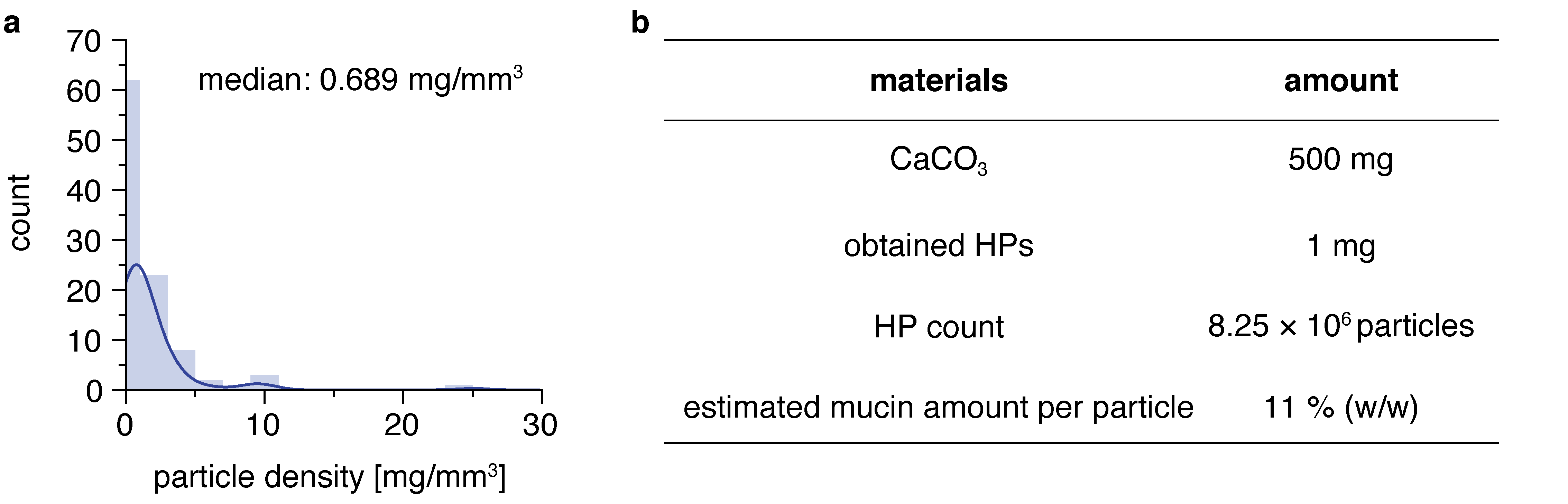 |
| --- |
| **Figure S7.** Density of the HPs (a) and the amount of materials used in the fabrication process to generate 1 mg of HPs (b). |

**8. Stability of the hollow PDA-mucin particles (HPs)**

PDA-mucin HPs were fabricated following the steps described in the methods section “*Fabrication of DM HPs*” of the main text. The HPs were then suspended in either D-PBS (pH 7.4) enriched with 20 mg/mL of bovine serum albumin (BSA; T844.2, Roth) which simulates synovial fluid,^[6]^ or in ddH_2_O (to simulate storage conditions). The samples containing HPs suspended in simulated synovial fluid were incubated at 37 °C with gentle shaking; the samples containing HPs suspended in ddH2O were stored at 4 °C without shaking. At each time point (*i.e*., day 0, day 2, day 5, day 7, day 14), the particle concentrations of the sample solutions were determined using a hemocytometer (Thoma), which was mounted onto a DMi8 Leica microscope and examined under phase contrast mode using a 20× objective. The size distributions of the particles were obtained by annotating the images of the particles and analyzing them using ImageJ Fiji. The obtained results are shown below.

| 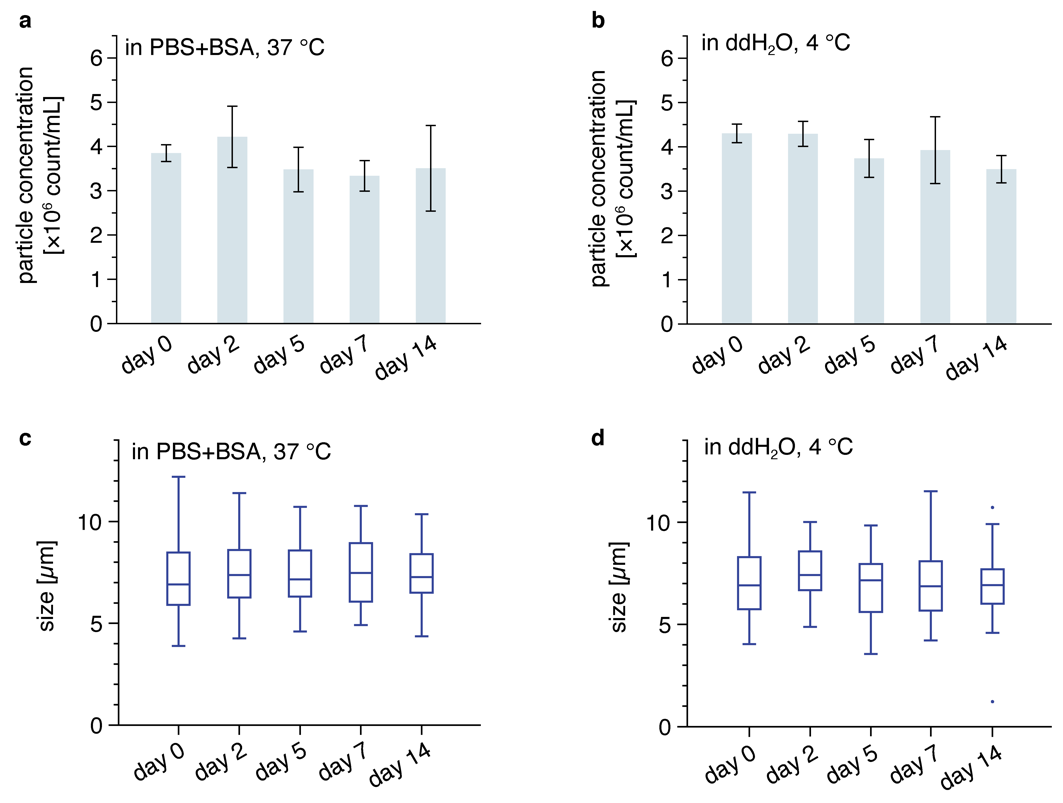 |
| --- |
| **Figure S8.** Stability of the HPs in a simulated physiological condition and in a storage condtion. (a, b) Particle concentrations after different time spans. The data is expressed as mean ± standard deviation. *n* = 3. (c, d) Size distributions of the HPs after different time spans. *n* = 50. |

**9. Fourier transform infrared (FTIR) spectroscopy measurements of the particles**

FTIR spectroscopy was carried out using a Nicolet iS50 instrument with a diamond-ATR module using a DTGS detector. New background spectra were recorded for each sample, and baseline correction was applied using the automatic correction function. All samples were characterized in a dry form. CaCO_3_ and lyophilized mucin were directly used for the measurements. PDA was prepared by shaking a dopamine solution (16 mg/mL in HEPES, pH 8.0) for 1.5 h at 37 °C. CaCO_3_@DM and HPs were prepared following the steps described in the methods section “*Fabrication of DM HPs*” of the main text. The obtained solutions were then lyophilized before the FTIR measurements.

| 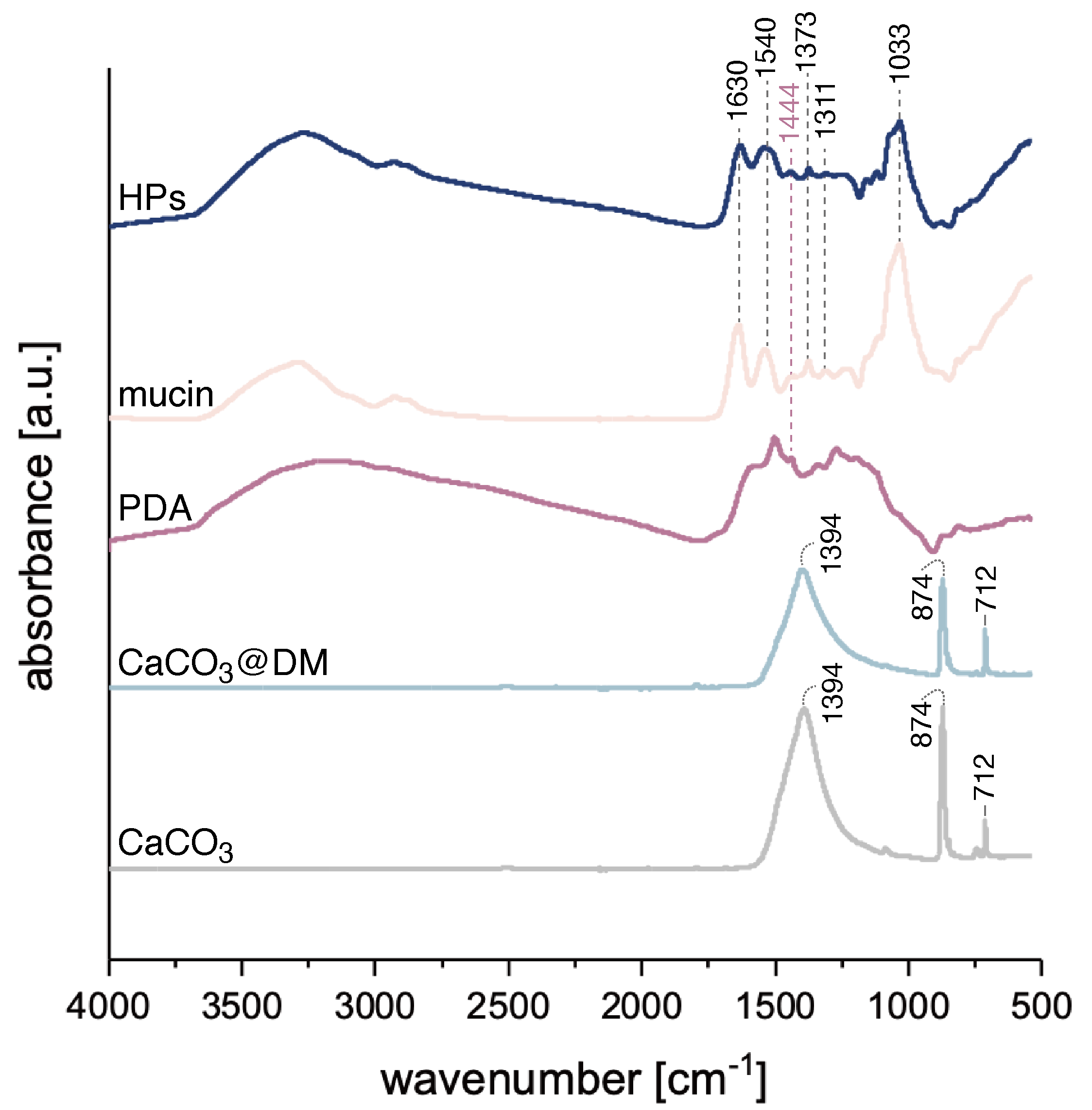 |
| --- |
| **Figure S9.** FTIR spectra of the samples, including CaCO_3_, CaCO_3_@DM, PDA, mucin, and HPs. |

**10. Size distribution of PDA-dextran HPs**

To fabricate PDA-dextran HPs, CaCO_3_ microparticles (average size: ~6 µm) were used as a template following the steps described in the main text. Bright field images of CaCO_3_ particles and PDA-dextran HPs (both suspended in ddH_2_O) were acquired on a DMi8 Leica microscope using a 63× lens. The diameters of the microparticles were then measured by annotating their bright field images using the software ImageJ Fiji (version: 2.9.0/1.53t).

| 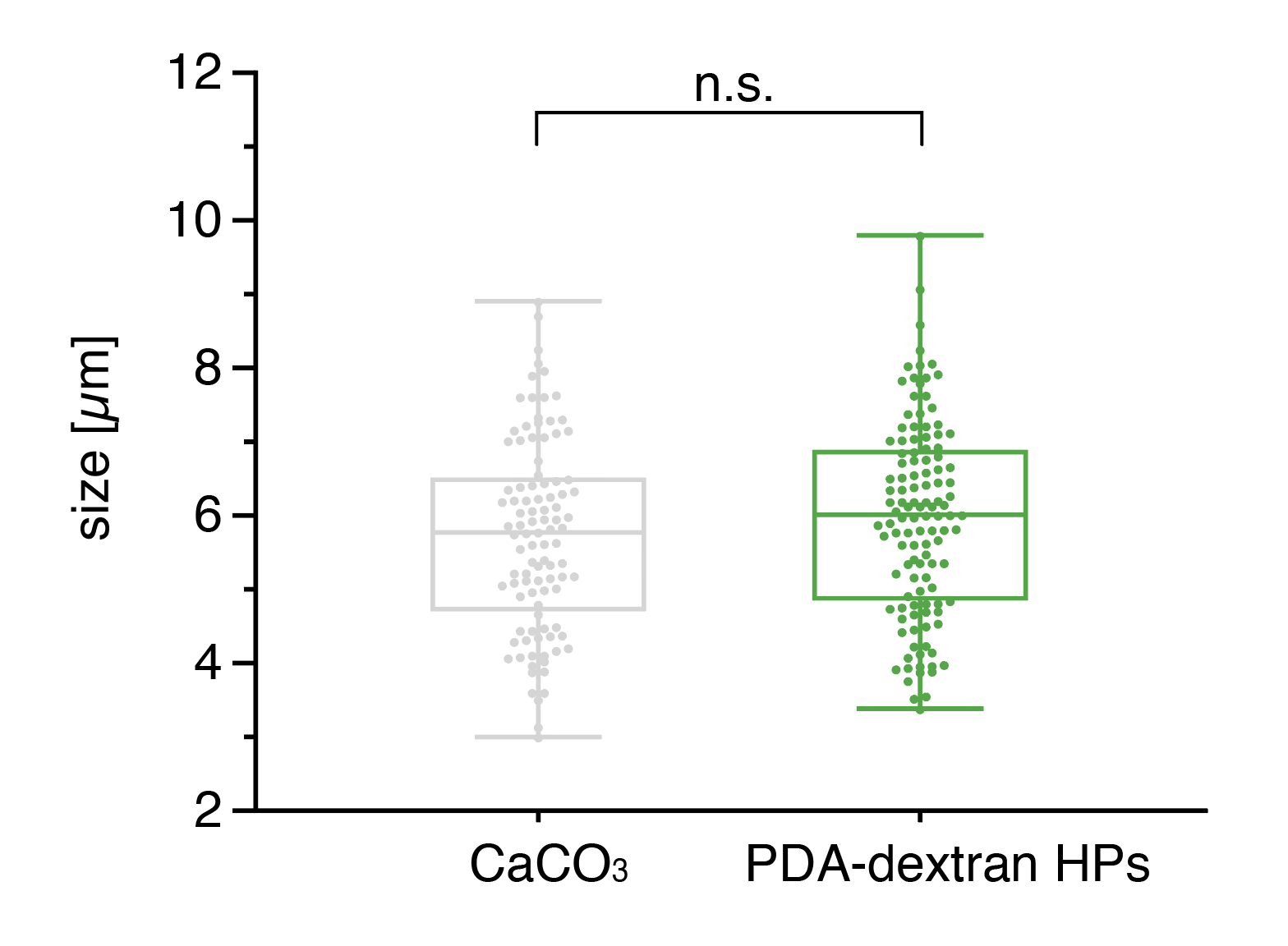 |
| --- |
| **Figure S10.** Size distributions of 6 µm CaCO_3_ particles and PDA-dextran HPs produced using these CaCO_3_ particles as templates (*n* ≥ 100). “n.s.” represents a nonsignificant difference determined in two-tailed t-tests based on a *p*-value of 0.05. |

**11. Loading positively charged cargo polymers into PDA-mucin HPs**

Fluorescein isothiocyanate-diethylaminoethyl-dextrans (FITC-DEAE-dextrans) with three different molecular weights (4 kDa, 70 kDa, 150 kDa) were purchased from Sigma Aldrich and used as model cargos for drug loading tests. To load these molecules into HPs, 50 µL of HPs (6.6 × 10^5^ particles/mL in ddH_2_O) was mixed with 50 µL of a FITC-DEAE-dextran solution (0.8 mg/mL in 20 mM HEPES, pH 7.0) and then incubated at room temperature for 30 min. Subsequently, the HPs (loaded with cargos) were washed with HEPES buffer for 3 times using centrifugation (2000 g, 2 min); afterwards, they were resuspended in HEPES buffer for imaging under a DMi8 Leica microscope in both phase contrast (PC) and fluorescence mode (excitation/emission wavelength: 495/519 nm) using a 63× objective.

| 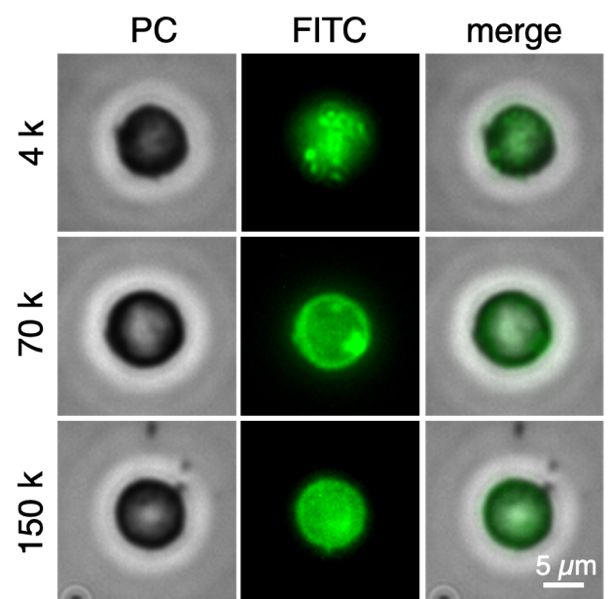 |
| --- |
| **Figure S11.** Phase contrast (PC) and fluorescence images of the HPs after encapsulating FITC-labeled DEAE-dextrans with molecular weights of 4 kDa, 70 kDa, and 150 kDa, respectively. |

**12. Permeability of the HPs**

To examine the permeability of HPs towards molecules with different molecular weights (MWs), the HPs were incubated with solutions containing FITC-CM-dextrans (0.4 mg/mL in 20 mM HEPES, pH 7.0). The MWs of the dextrans were 4, 70, or 150 kDa. To visualize the locations of the FITC-CM-dextrans after 1 h of incubation, the mixtures were examined using fluorescence microscopy. In detail, the samples were added onto glass slides and covered with cover slips and then visualized on a DMi8 Leica microscope in both phase contrast (PC) and fluorescence mode (excitation/emission wavelength: 495/519 nm) using a 63× objective. The obtained fluorescence images were analyzed using ImageJ Fiji, where a horizontal line was drawn on each image, followed by obtaining the fluorescence intensity at different locations of the drawn lines.

| 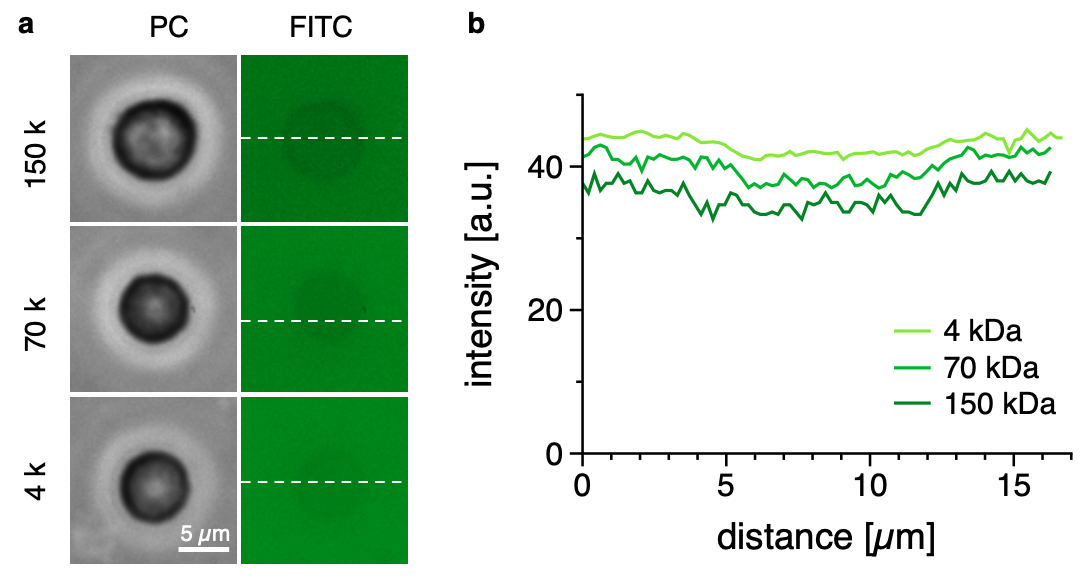 |
| --- |
| **Figure S12.** Permeability of the HPs towards FITC-CM-dextrans with different molecular weights. (a) Phase contrast (PC) and fluorescence images of the HPs when incubated with FITC-CM-dextrans with a molecular weight of 4 kDa, 70 kDa, and 150 kDa, respectively. The fluorescence intensity along the dashed lines in the fluorescence images is shown in (b). “Distance” indicates how far away the measured point is located from the left end of the lines. |

**13. Loading capacity of HPs with different “locks”**

FITC-CM-dextrans with three different molecular weights (*i.e.*, 4, 70, 150 kDa) were used as model cargos for drug loading tests following the steps described in the methods section “*Drug loading into HPs*”. The cargo loading capacity was then obtained (**Figure S13**) based on the following equation:

$$loading capacity \left[ wt\% \right]=\left( \frac{weight of loaded dextrans \left[ \mu g \right]}{weight of loaded dextrans+weight of the particles \left[ \mu g \right]} \right)\times100$$

| **** |
| --- |
| **Figure S13.** Loading capacity of HPs with different “locks”. FITC-CM-dextrans with different molecular weights (*i.e.*, 4, 70, 150 kDa) are used as model cargos. The tested “locks” include lectin, Ca^2+^, PDL, and Ag+. HPs without any “lock” are used as a control group (*n* = 3). The data is expressed as mean ± standard deviation. |

**14. Interactions between silver ions and PDA/mucin molecules**

The size distribution of the samples was obtained using a Litesizer 500 Zetasizer (Anton Paar GmbH) in dynamic light scattering (DLS) mode. To prepare PDA solutions, a 16 mg/mL dopamine solution prepared in HEPES buffer (20 mM, pH 8.0) was shaken for 1.5 h at 37 °C to allow for the formation of PDA. Then, the obtained PDA solution was mixed with HEPES buffer (5 mM, pH 7.0) containing either NaCl or AgNO_3_, with a final concentration of 0.1 mg/mL for PDA and 40 mM for a salt (*i.e*., NaCl or AgNO_3_). Similarly, to prepare mucin solutions, a mucin stock solution (8 mg/mL) was added to HEPES buffer (5 mM, pH 7.0) containing either NaCl or AgNO_3_, with a final concentration of 0.1 mg/mL for mucin and 40 mM for a salt (*i.e.*, NaCl or AgNO_3_). Here, NaCl solutions were used as a control group to account for the effect of ionic strength brought about by AgNO_3_ on the size distribution of the molecules. Afterward, 950 µL of each solution was added into a disposable four-clear-sided cuvette, which was then inserted into the device for measurements. For the DLS measurements, the back scatter (175°) mode was used for PDA solutions (containing NaCl or AgNO_3_), whereas side scatter (90°) mode was used for mucin solutions (containing NaCl or AgNO_3_), which were chosen based on the transparency of the solutions. The intensity-weighted size distribution of each sample was then obtained **(Figure S14)**.

| 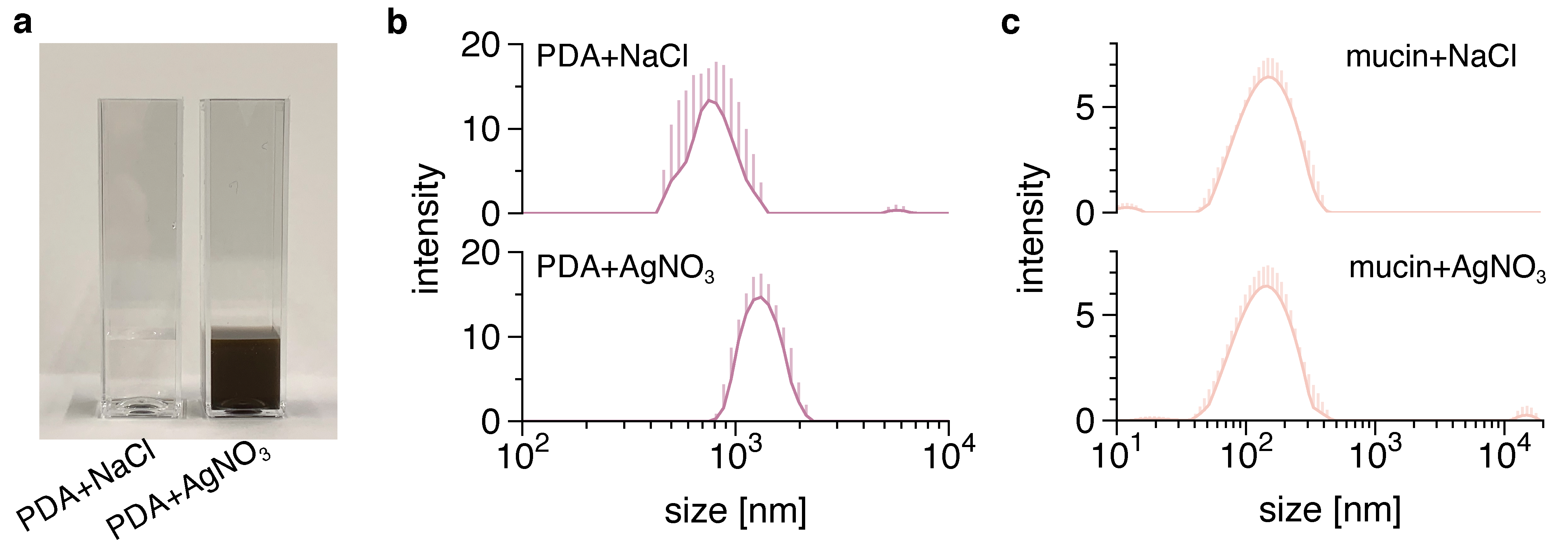 |
| --- |
| **Figure S14**. Interactions between silver ions and PDA/mucin. (a) Images of mixed PDA (0.1 mg/mL) solutions. Left: a solution containing PDA and NaCl; right: a solution containing PDA and AgNO_3_. (b) Size distribution of PDA molecules when mixed with either NaCl or AgNO_3_. (c) Size distribution of mucin molecules when mixed with either NaCl or AgNO_3_. *n* = 3. The data is expressed as mean ± standard deviation. |

**15. Free radical scavenging efficiency of PDA and mucin solutions**

Two assays were employed to test the free radical scavenging capabilities of PDA and mucin solutions, *i.e.*, a 1,1-diphenyl-2-picrylhydrazyl (DPPH•) assay and a 2,2′-azino-bis(3-ethylbenzothiazoline-6-sulfonic acid) diammonium salt radical cations (ABTS•^+^) assay (following the steps described in the main text). Here, after being mixed with the DPPH• solution or the ABTS•^+^ solution, the final concentrations of PDA or mucin in the solutions (prepared in ddH_2_O)

were 0, 1.56, 3.12, 6.25, 12.5, or 25 µg/mL.

| 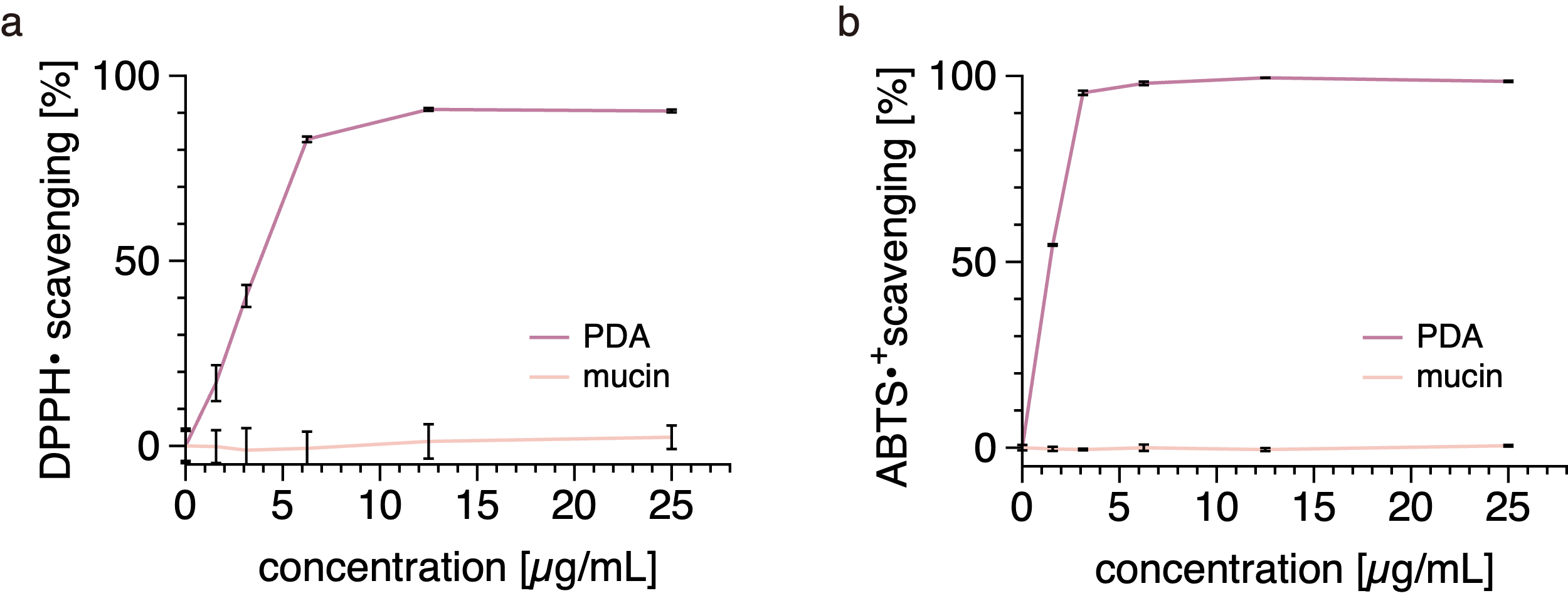 |
| --- |
| **Figure S15.** The scavenging efficiency of (a) DPPH• and (b) ABTS•+ when treated with PDA or mucin in different concentrations (*n* = 3). The data is expressed as mean ± standard deviation. |

**16. Intracellular ROS scavenging capability of PDA and mucin**

To test the ROS levels in the cells, 2',7'-dichlorodihydrofluorescein diacetate (DCFH-DA; HY-D0940, MCE®) was used to stain the cells. To do so, the HeLa cells were seeded into a 96-well plate (5,000 cells/well) and incubated overnight. Afterwards, the cells were incubated with DCFH-DA (10 µM in D-PBS) for 0.5 h, and then washed twice with D-PBS (containing 0.9 mM CaCl_2_ and 0.5 mM MgCl_2_). Subsequently, the cells were incubated with different solutions including i) pure cell culture medium, ii) medium containing H_2_O_2_ (400 µM), iii) medium containing H_2_O_2_ (400 µM) and PDA (100 µg/mL), iv) medium containing H_2_O_2_ (400 µM) and mucin (100 µg/mL). After 55 min of incubation, the cells were imaged on a DMi8 Leica microscope in both, phase contrast and fluorescence mode (excitation/emission wavelength: 495/519 nm) using a 10× objective (N PLAN 10×/0.25 DRY). The obtained images are shown in **Figure S16**.

| **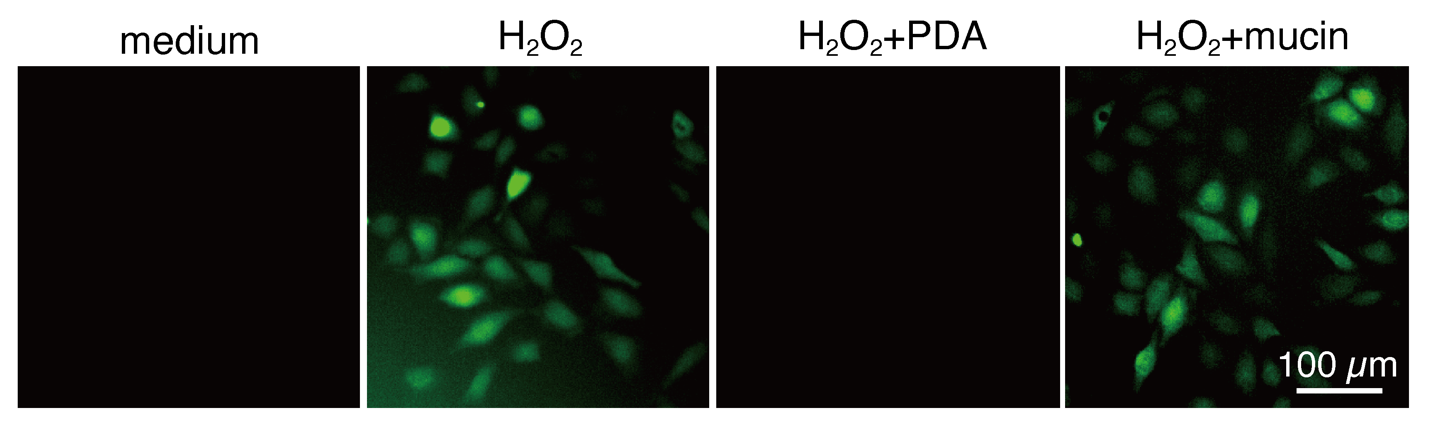** |
| --- |
| **Figure S16.** Fluorescence images of HeLa cells stained with the dye DCFH-DA to visualize intracellular ROS (green signal). HeLa cells are incubated with different solutions, *i.e*., pure medium, medium containing H_2_O_2_, medium containing H_2_O_2_ and PDA, and medium containing H_2_O_2_ and mucin. The scale bar in the last image applies to all images in this figure. |

**17. Tissue adhesion properties of pure PDA particles and pure mucin molecules**

To label PDA with ATTO-594 (ATTO-TEC GmbH), a dopamine solution (16 mg/mL; prepared in 20 mM HEPES, pH 8.0) enriched with 0.1 mg/mL of ATTO-594 was shaken at 37 °C for 2 h. Then, the formed PDA particles labelled with ATTO-594 were collected and washed with ddH_2_O using centrifugation (7800 rpm, 5 min). To label mucin molecules with ATTO-594, a carbodiimide coupling method was used following the steps described in the methods section “*Fluorescence imaging of the particles*” of the main text. Subsequently, tissue adhesion tests using the PDA solutions or mucin solutions were conducted on porcine cartilage or tissue pieces from the dorsal surface of the porcine tongue following the steps described in the methods section “*Ex vivo particle adhesion tests to tissues*” of the main text. The obtained images are shown in **Figure S17**.

| 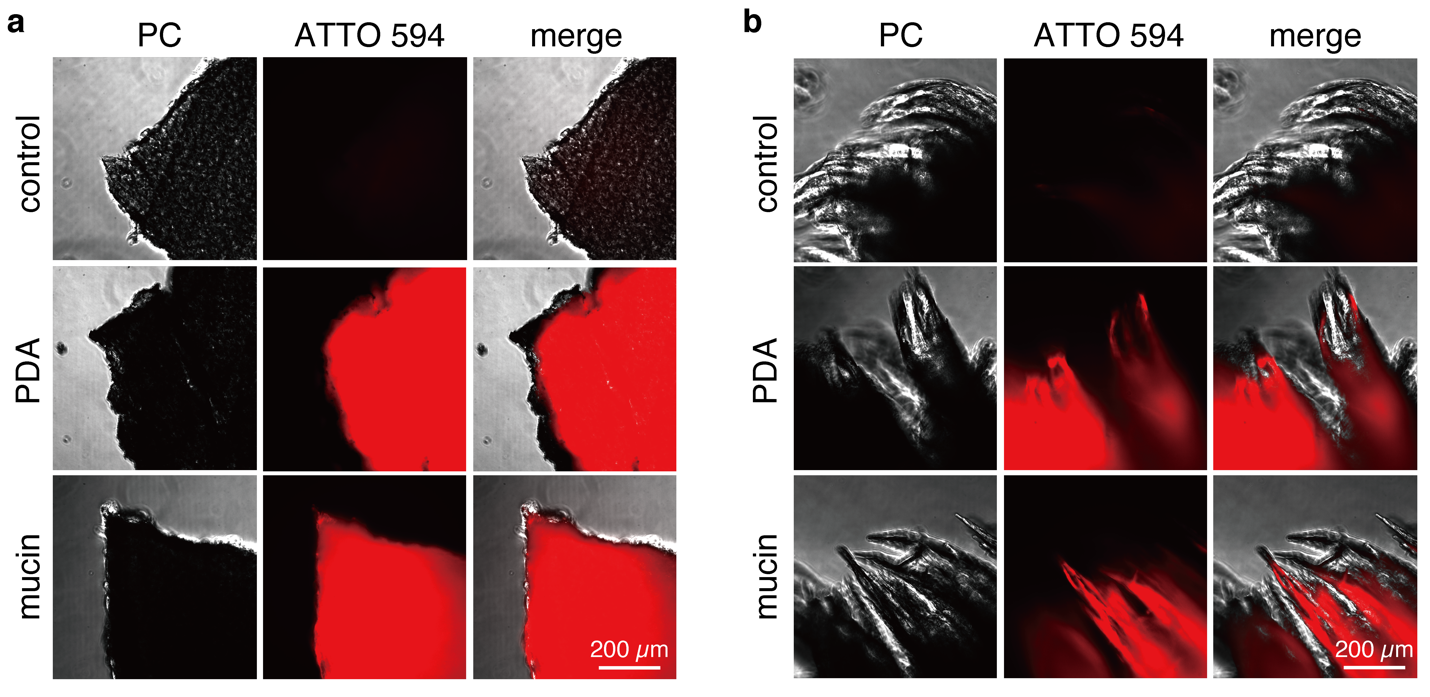 |
| --- |
| **Figure S17.** Phase contrast (PC) and fluorescence images of (a) cartilage pieces and (b) tongue tissue samples, respectively, after incubation with PDA-ATTO-594 or mucin-ATTO-594. As controls, tissue samples are incubated with a buffer devoid of those fluorescently-labelled PDA and mucin molecules. |

**18. Lubricity of pure PDA and pure mucin solutions**

The lubrication performance of pure PDA and pure mucin solutions was investigated using a commercial shear rheometer (MCR 302, Anton Paar, Austria) following the steps mentioned in the methods part “*Rotational Tribology*” of the main text. For each measurement, 500 µL of a PDA or mucin solution (1 mg/mL in 20 mM HEPES, pH 7.0) was used; HEPES (20 mM, pH 7.0) buffer served as a control.

| **** |
| --- |
| **Figure S18.** Friction curves obtained on PDMS when employing different solutions as lubricants. Values shown denote mean values, error bars represent the standard deviation as determined from *n* = 3 independent experiments. |

**19. Calibration curves for the dextran release tests**

FITC-carboxymethyl-dextran (FITC-CM-dextran, 4 kDa, at a concentration of 2.5, 5, or 10 µg/mL) was dissolved in different solvents including ddH_2_O, PBS (pH 7.4), PBS with 10 mM H_2_O_2_ (pH 7.4), and PBS with 8 mg/mL glucose (pH 7.4), followed by incubation for different time spans at 37 °C. Then, 100 µL of each solution was transferred to a black 96-well plate, and 100 µL of D-PBS (pH 7.4) was added to each well (D-PBS was supplemented here since FITC exhibits a lower fluorescence intensity in pure ddH_2_O). The fluorescence intensity (FI) of each sample was determined using a plate reader at an excitation/emission wavelength of 495/520 nm.

| **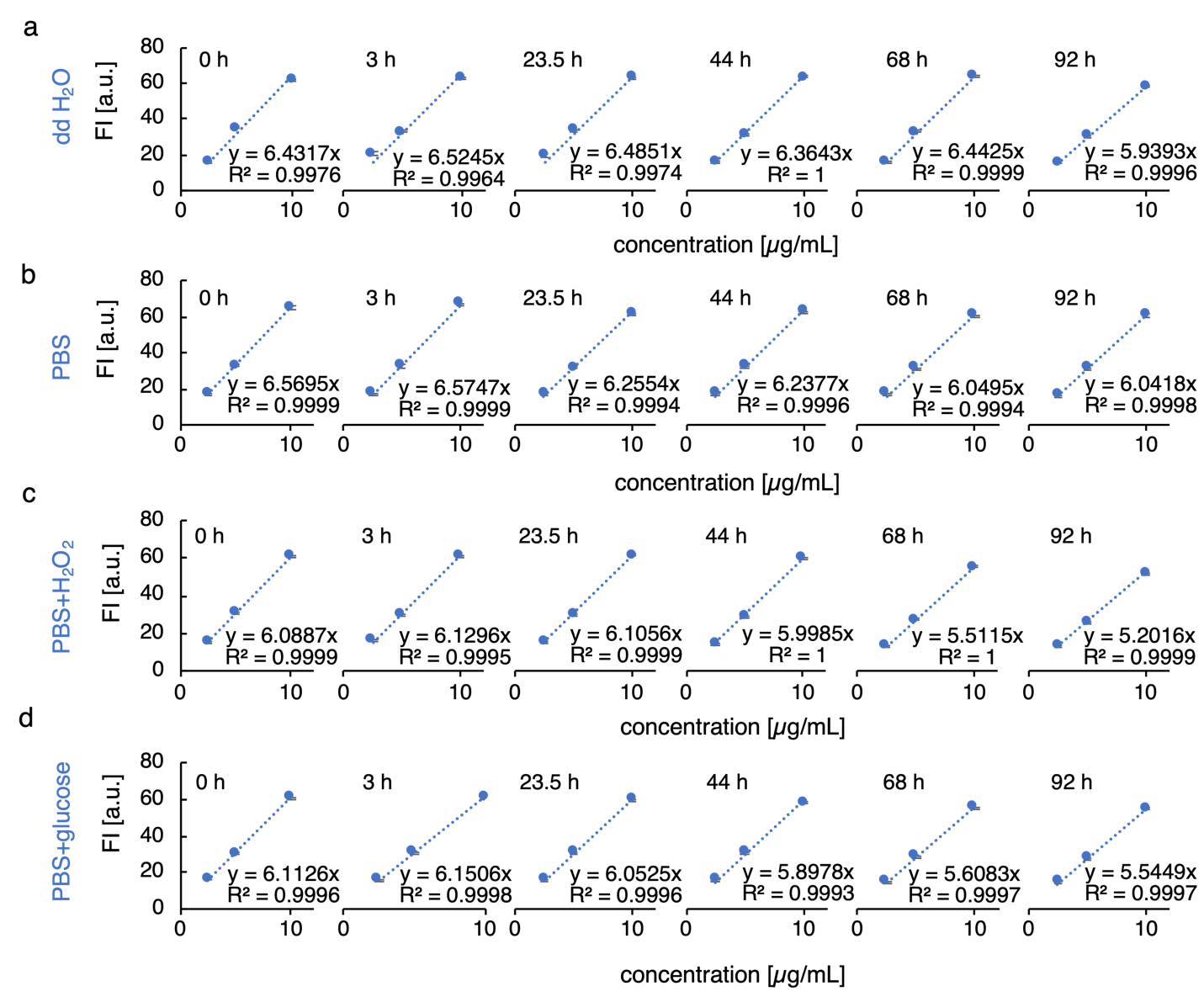** |
| --- |
| **Figure S19.** Calibration curves obtained for FITC-CM-dextran (4 kDa) dissolved in different solvents including (a) ddH_2_O, (b) PBS, (c) PBS with H_2_O_2_, and (d) PBS with glucose. The calibration curves were acquired after different incubation times at 37 °C; for each condition, *n* = 3. The data is expressed as mean ± standard deviation. |

**References**

1. Asha, A. B.; Chen, Y.; Narain, R., “Bioinspired dopamine and zwitterionic polymers for non-fouling surface engineering,” *Chemical Society Reviews* *50*, no. 20 (2021): 11668-11683. <https://doi.org/10.1039/D1CS00658D>

2. Marczynski, M.; Balzer, B. N.; Jiang, K.; Lutz, T. M.; Crouzier, T.; Lieleg, O., “Charged glycan residues critically contribute to the adsorption and lubricity of mucins,” *Colloids and Surfaces B: Biointerfaces* (2020): 110614, <https://doi.org/10.1016/j.colsurfb.2019.110614>

3. Xu, Y.; Hu, J.; Hu, J. et al., “Bioinspired polydopamine hydrogels: Strategies and applications,” *Progress in Polymer Science* (2023): 101740, <https://doi.org/10.1016/j.progpolymsci.2023.101740>

4. Ballester, B.; Milara, J.; Cortijo, J., “Mucins as a New Frontier in Pulmonary Fibrosis,” *Journal of Clinical Medicine* *8*, no. 9 (2019): 1447. <https://doi.org/10.3390/jcm8091447>

5. Fan, D.; Miller Naranjo, B.; Mansi, S.; Mela, P.; Lieleg, O., “Dopamine-Mediated Biopolymer Multilayer Coatings for Modulating Cell Behavior, Lubrication, and Drug Release,” *ACS Applied Materials & Interfaces* *15*, no. 31 (2023): 37986-37996. <https://doi.org/10.1021/acsami.3c05298>

6. Thing, M.; Mertz, N.; Ågårdh, L.; Larsen, S. W.; Østergaard, J.; Larsen, C., “Simulated synovial fluids for in vitro drug and prodrug release testing of depot injectables intended for joint injection,” *Journal of Drug Delivery Science and Technology* (2019): 169-176, <https://doi.org/10.1016/j.jddst.2018.11.012>
